# Phosphonate- and Phosphonic Acid-Functionalized Polycyclooctenes Enabling High Ionic Conductivity and Intrinsic Flame Retardancy in Solid-State Lithium-Ion Batteries

**DOI:** 10.1021/acsomega.6c02084

**Published:** 2026-06-08

**Authors:** Muhammad Syukri Mohamad Misenan, Aze Ilgın Gündoğdu, Maheen Rahim, Aysel Kantürk Figen, Tarık Eren

**Affiliations:** † Department of Chemistry, School of Arts and Science, 52999 Yildiz Technical University, Istanbul 34220, Turkiye; ‡ Department of Chemical Engineering, Faculty of Chemistry and Metallurgy, Yıldız Technical University, İstanbul 34220, Turkiye; § BATLAB Research Center, Clean Energy Technologies Institute, Yıldız Technical University, Istanbul 34220, Turkiye

## Abstract

Polymer electrolytes are considered promising materials for next-generation solid-state lithium-ion batteries due to their enhanced safety, flexibility, and electrochemical stability compared to conventional liquid electrolytes. Nevertheless, major bottlenecks including low room-temperature ionic conductivity, poor electrode/electrolyte interfacial stability, and limited flame-retardant properties still hinder their practical applications. In the pursuit of safer and high-performance lithium-ion batteries (LIBs), polymer electrolytes with improved ionic conductivity, thermal stability, and flame retardancy are highly desirable. In this study, a novel phosphonate-functionalized polycyclooctene (PPCO) was synthesized and characterized as a solid polymer electrolyte for LIBs. The synthetic route began with a thiol–ene click reaction between cyclooctadiene and mercaptoethanol to afford a hydroxyl-functionalized monomer, which was subsequently reacted with a chlorophosphate reagent to introduce phosphonate groups. Polymerization using Grubbs’ third-generation catalyst yielded a polymer bearing pendant phosphonate functionalities, PolyCOD_phosphonate_. Phosphonic acid derivatives, PolyCOD_phosphonic acid_, were further prepared via treatment with trimethylsilyl bromide. These polymers were blended with polyvinylidene difluoride (PVDF) and lithium bis­(trifluoromethanesulfonyl)­imide (LiTFSI). Thermal analysis confirmed successful functionalization and high thermal stability (>300 °C). Microcone calorimetry revealed that PolyCOD_phosphonic acid_ exhibited superior heat release rate (HRR) reduction compared to PolyCOD_phosphonate_. Electrochemical impedance spectroscopy demonstrated enhanced lithium-ion conductivity in the presence of LiTFSI, attributed to the strong solvation ability of the phosphonate moieties. A maximum conductivity of 0.63 × 10^–3^ S cm^–1^ at ambient temperature was achieved. The incorporation of phosphonate functionalities not only improves ionic transport but also imparts flame-retardant characteristics, establishing these materials as promising candidates for next-generation solid-state LIB electrolytes. Future work will include Li^+^ transference number determination, extended electrochemical stability analysis, and coin cell testing to evaluate cycling performance and interfacial stability, alongside further optimization of polymer structure for enhanced ionic conductivity.

## Graphical abstract

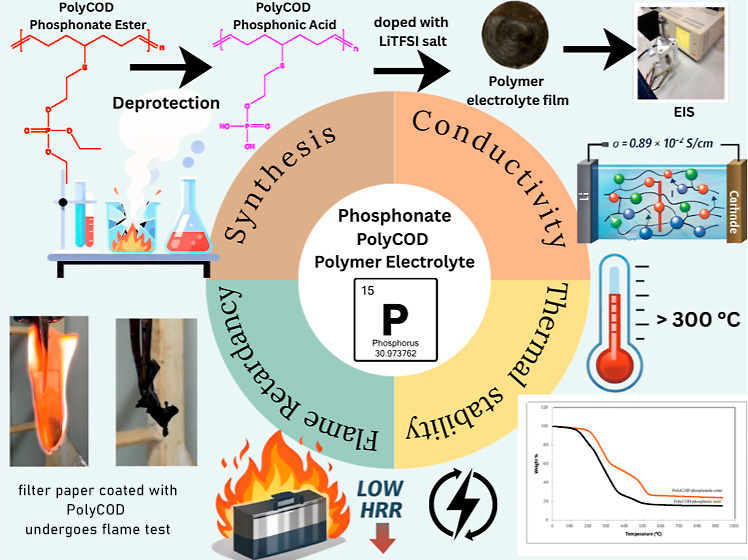

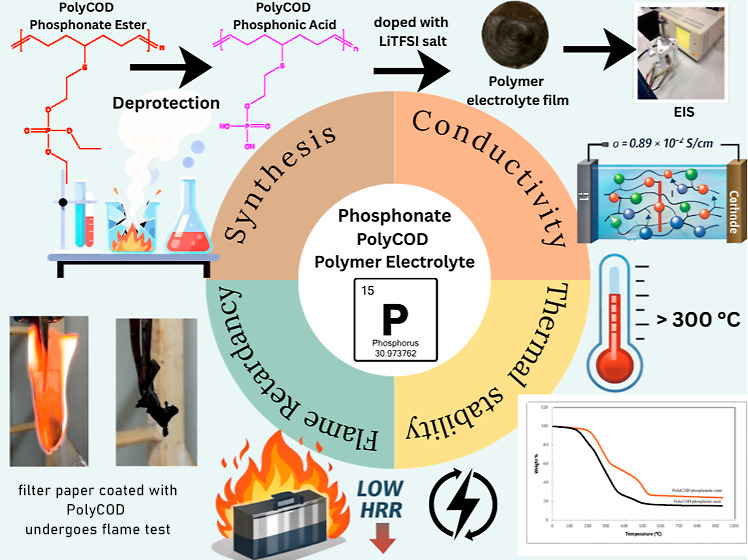

## 1 Introduction

Batteries are expected to play a crucial and indispensable role in energy storage applications, particularly in electric vehicles (EVs) and portable electronic devices, within the rapidly advancing technological landscape.^1^ ^,^ ^2^ Battery Electric Vehicles (BEVs) mark a significant transformation in the automotive sector,^3^ paving the way for the emergence of new automakers.^4^ While it remains uncertain which of these newcomers will achieve long-term financial sustainability,^5^ the number of BEV manufacturers continues to grow.^6,7^ Historically, the country’s automotive industry has been dominated by a small and relatively stable group of large original equipment manufacturers (OEMs). These firms are typically headquartered in a limited number of core automotive countries, which not only host the headquarters of major OEMs but also account for much of the industry’s global production and research and development (R&D) activities. In recent years, a growing share of this R&D effort has focused on advanced battery technologies, reflecting the central role of electrification in the future of mobility. Battery research has become a strategic priority for OEMs as they seek to enhance energy density, safety, and lifecycle performance while reducing costs and environmental impacts. The integration of local battery innovation and manufacturing capabilities is now considered a key factor in strengthening national competitiveness within the evolving global automotive landscape.

Broadly, batteries are classified into two categories: primary and secondary. Primary batteries are nonrechargeable, whereas secondary batteries can be recharged. Among these, lithium-ion batteries (LIBs) are classified as secondary batteries and offer several advantages, including low self-discharge rate, long cycle life, high operating voltage, and high energy density.^8^ Conventional LIBs operate based on the Li^+^ intercalation mechanism.^9^ However, their relatively low charge capacity poses challenges for applications that demand higher energy storage, such as aerospace, electric vehicles, and hybrid electric vehicles.^10^ To date, the energy density of conventional LIBs has reached approximately 260 Wh kg^–1^, which represents a limiting factor for further advancements. Therefore, addressing the key limitations of conventional lithium-ion batteries is essential for advancing technology. In addition to their restricted energy density, traditional LIBs face several other challenges, including commercialization barriers, volume expansion, particle surface reconstruction, electrolyte decomposition, solid–electrolyte interphase (SEI) formation, and phase transitions.^11^ Research indicates that next-generation lithium-ion batteries with higher energy densities hold strong potential for diverse applications. Such batteries are expected to offer advantages such as reduced cost, increased payload capacity, and lower toxicity.^8^ As an alternative to intercalation-type anodes, replacing graphite with lithium–metal anodes has the potential to overcome the limitations of energy density. However, this approach presents significant safety concerns when used with conventional liquid electrolytes.^12^ Plating/stripping tests further reveal the nonuniform distribution of Li^+^ ions, which promotes dendrite formation. Lithium-ion conductive polymer electrolytes (PEs) have emerged as promising candidates to simultaneously enhance energy density and safety.^13^ ^,^ ^14^ In particular, solid polymer electrolytes can effectively suppress dendrite initiation and growth.^15–24^ Unlike traditional liquid electrolytes, these polymer-based systems are also nonflammable.^25^

Polycyclooctene (PCO) can be synthesized via ring-opening metathesis polymerization (ROMP).^26^ Its unique molecular architecture imparts a combination of elastomeric properties and tunable crystallinity, making it a material of interest for various advanced applications. The presence of unsaturated bonds in the polymer backbone allows for postpolymerization modifications, enabling the introduction of functional groups that can tailor the material’s properties for specific uses. The functionalization of PCO has expanded its applicability in the field of polymer electrolytes. By grafting poly­(ethylene glycol) (PEG) chains onto the PCO backbone, researchers have developed amphiphilic copolymers that form continuous PEG channels, enhancing ionic conductivity. Such materials have shown promise in the development of solid polymer electrolyte membranes for energy storage devices.^27^ Additionally, PCO-based polymeric ionic liquids (PILs) represent a complementary and equally versatile platform in which the ROMP-derived PCO backbone provides a soft, flexible, low-*T*_g_ scaffold with extensive postfunctionalization potential due to its high density of pendant C=C bonds.^28^ The versatility of PCO-based polymers, stemming from their modifiable structure and favorable mechanical properties, positions them as promising candidates for applications in smart materials,^29^ energy storage,^30^ and tissue engineering.^31^

Incorporating flame retardants into polymer electrolytes is an effective approach to enhancing the safety of lithium metal batteries (LMBs).^32^ However, these additives often lead to reduced ionic conductivity and poor electrode compatibility, triggering internal reactions that hinder the formation of a stable solid electrolyte interface (SEI) and consequently degrade the cycling performance.^33^ Reactive-type flame retardants have been proposed to overcome the aforementioned challenges.^34,35^ Phosphorus-based flame retardants have emerged as promising alternatives owing to their strong fire suppression efficiency.^36^ Materials such as ammonium polyphosphate (APP), aluminum phosphate (AlPO_4_), and melamine polyphosphate (MPP) are commonly employed as additives in battery systems to enhance thermal and flame resistance.^37^ In contrast to halogen-based flame retardants, phosphorus-based systems provide notable environmental and safety benefits, including lower toxicity and reduced smoke release during combustion.^38^ Consequently, phosphorus-based flame-retardant compounds are predominantly employed as additives rather than solvents, enabling their incorporation at relatively low loadings. This approach preserves the intrinsic properties of the electrolyte while simultaneously reducing the material cost and mitigating adverse effects on electrochemical performance.^39^

In this study, novel phosphonate- and phosphoric acid-functionalized polycyclooctene homopolymers were successfully synthesized via ROMP. The synthetic route began with the preparation of a cyclooctene-based monomer, derived from cyclooctadiene and mercaptoethanol through a thiol–ene click reaction. This reaction introduced a terminal hydroxyl group onto the monomer framework, providing a reactive site for further functionalization. Subsequently, the phosphonate moiety was introduced by reacting the hydroxyl-functionalized monomer with diethyl chlorophosphate. The resulting monomer was then polymerized using Grubbs’ third-generation catalyst under inert conditions to afford the desired polycyclooctene-based polymer bearing pendant phosphonate ester groups, PolyCOD_phosphonate_ (Scheme 1). Then the PolyCOD_phosphonate_ underwent the deprotection process by trimethyl bromo silane to produce a phosphonic acid-based polymer, PolyCOD_phosphonic acid_ (Scheme 2). Subsequently, the conductivity, flame-retardant performance, and thermal properties of the polymers were investigated. This synthetic strategy combines the efficiency of ROMP, yielding a polymer with potential applications in ion-conductive membranes and inherent flame-retardant properties, which are an important requirement for LIBs.^40^ ^,^ ^41^

**Scheme 1:**
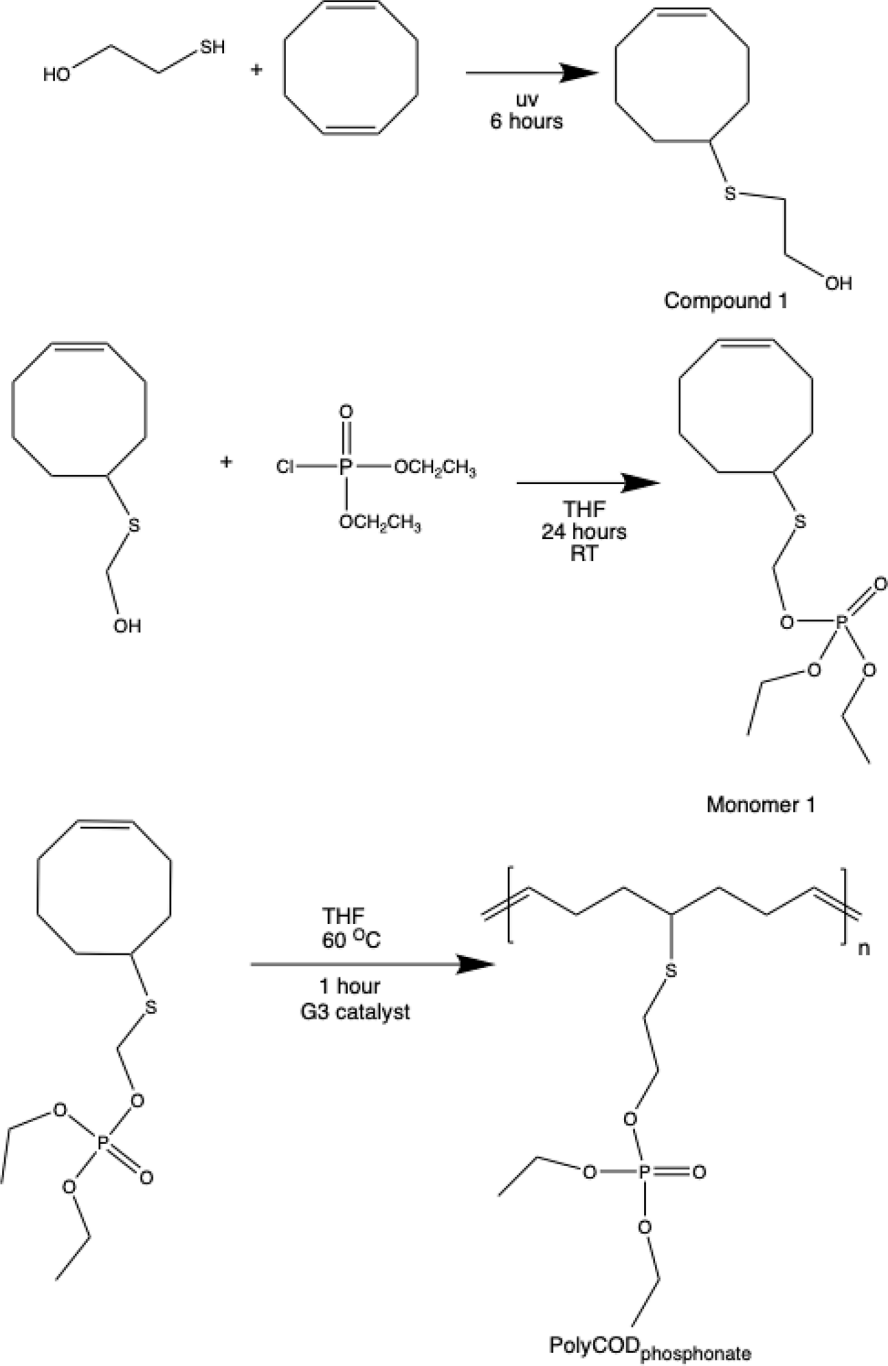
Monomer and Synthesis Route of PolyCOD_phosphonate_

**Scheme 2:**
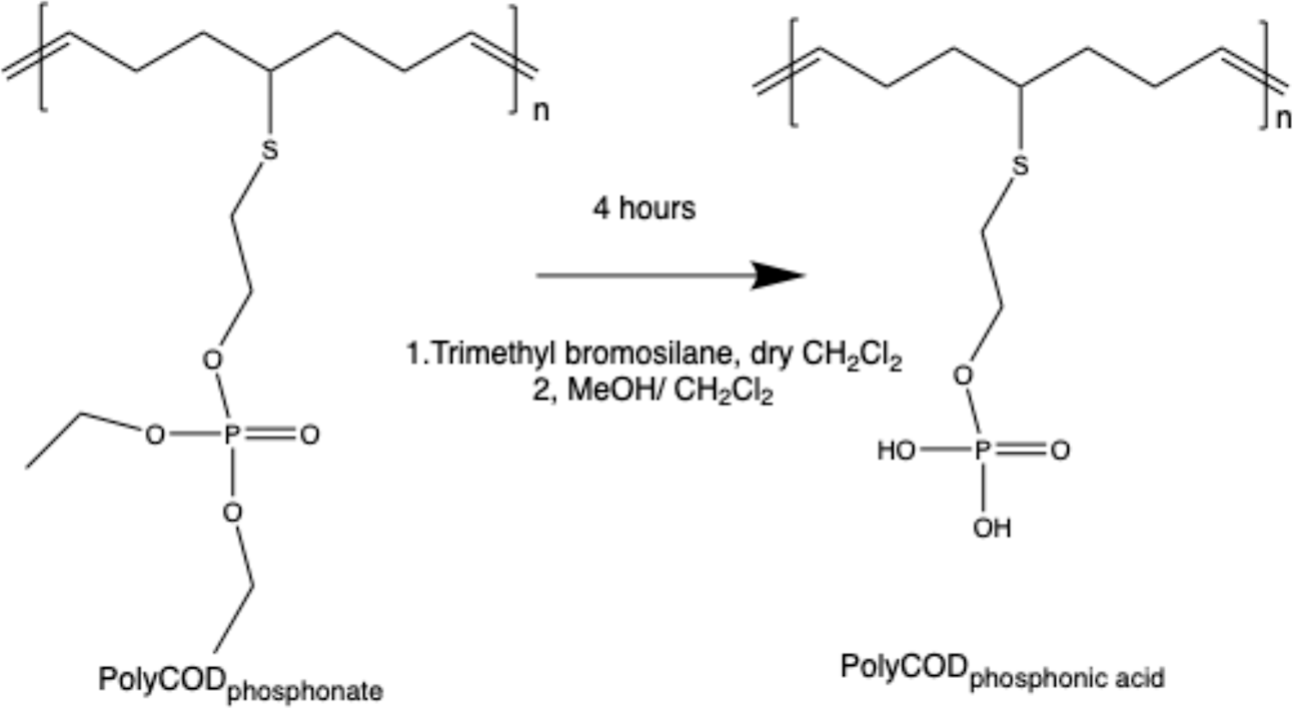
Synthesis of PolyCOD_phosphonic acid_

## 2 Experimental Section

PolyCOD_phosphonate_ was synthesized via a thiol–ene click reaction followed by ROMP using Grubbs’ third-generation catalyst. PolyCOD_phosphonic acid_ was obtained by deprotection of PolyCOD_phosphonate_ (see Supporting Information for full experimental details). Polymer electrolytes were prepared by solution casting with PVDF and LiTFSI and characterized by Fourier Transform Infrared (FTIR), Nuclear Magnetic Resonance (NMR), Gel Permeation Chromatography (GPC), Thermogravimetric Analysis (TGA), Differential Scanning Calorimetry (DSC), Microscale Combustion Calorimetry (MCC), Electrical Impedance Spectroscopy (EIS), Cyclic Voltammetry (CV), and Qualitative Burn Tests.

## 3 Result and Discussion

### 3.1 Monomer (M1) Synthesis

The synthesis of the phosphonate-based monomer was carried out in two steps. First, mercaptoethanol was added to cyclooctadiene via a UV light-induced thiol–ene reaction (Scheme 1). After purification by column chromatography, the product (Compound 1) was reacted with commercially available diethyl chlorophosphate to obtain the target monomer (M1). In TLC analysis, the *R*_f_ values for Compound 1 and the monomer were 0.68 and 0.23, respectively (Figure S1).

The Fourier Transform Infrared (FTIR) spectrum of Compound 1 (Figure 1) displays a broad absorption band centered at approximately 3360 cm^–1^, which is indicative of O–H stretching vibrations. This signal is typically associated with the presence of hydroxyl groups, such as those found in alcohols or phenolic compounds; in this case, the signal arises from mercaptoethanol. The broad nature of the peak may further suggest the involvement of hydrogen bonding. A notable absorption band is also observed at 1640 cm^–1^, corresponding to the C=C stretching vibration from cyclooctadiene. This peak is characteristic of alkene groups, which confirms the presence of carbon–carbon double bonds within the molecular structure.

**Figure 1:**
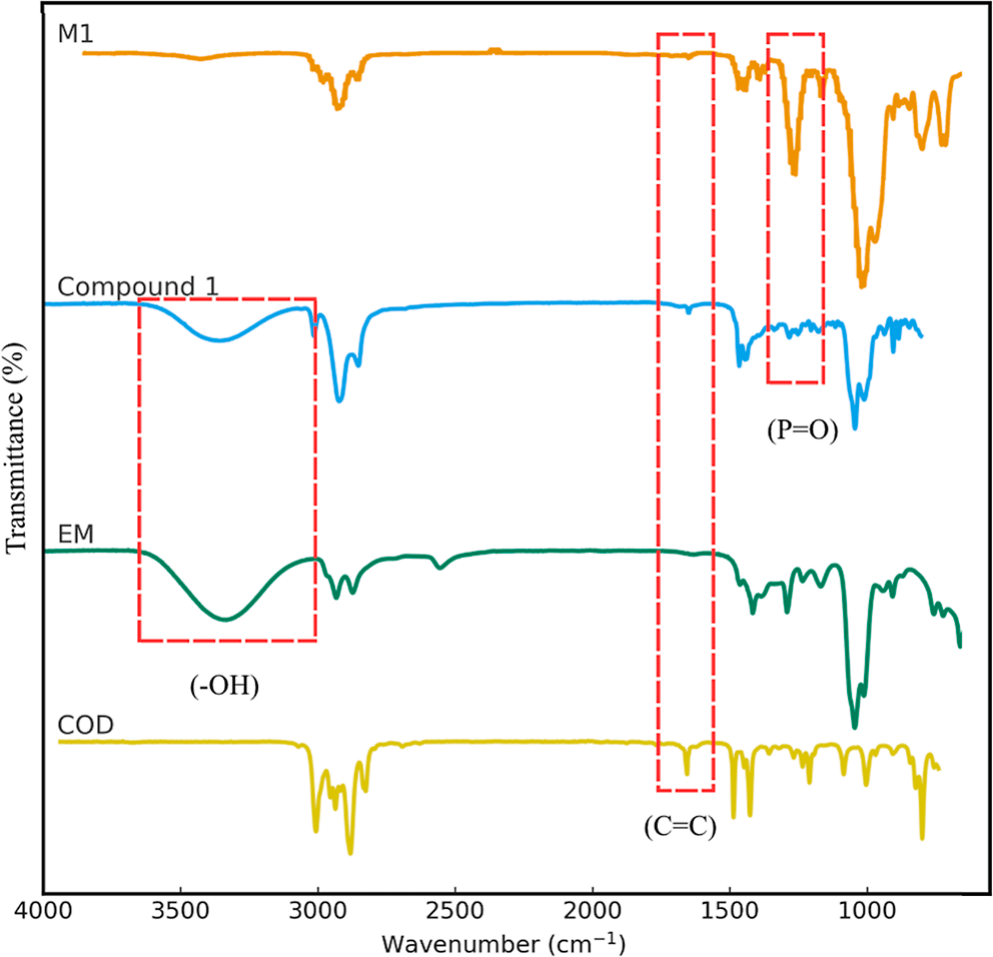
FTIR spectra of cyclooctadiene (COD), mercaptoethanol (EM), Compound 1, and phosphonate monomer (M1).

Compound 1 was then reacted with diethyl chlorophosphate to obtain phosphonate functional cyclooctene monomer M1. The FTIR spectrum of sample M1 exhibits the disappearance of the broad O–H stretching band previously cantered around 3360 cm^–1^, confirming the reaction of hydroxyl groups with diethyl chlorophosphate. A prominent absorption band at 1250 cm^–1^ is attributed to the P=O stretching vibration, indicative of successful phosphorylation, presumably associated with the cyclooctadiene moiety.

The ^1^H NMR spectrum confirmed the cyclooctadiene, Compound 1, and Monomer 1 structure, respectively (Figure 2). First, Compound 1 showed a characteristic mercapto thiol functional group. The presence of vinylic protons from cyclooctadiene was evidenced in the region of 5.5–5.7 ppm. A distinct peak at 3.6 ppm corresponded to the methylene protons adjacent to the hydroxyl (–OH) group, while the hydroxyl proton itself appeared as a broad signal at 3.7 ppm. The ^1^H NMR spectrum presented in Figure 2c provides clear evidence for the successful completion of the reaction between the synthesized Compound 1 and diethyl chlorophosphate. Notably, the appearance of new signals in the spectrum around 1.25 and 4.10 ppm is indicative of the incorporation of the diethyl chlorophosphate moiety into the polymer backbone. These peaks correspond to the methyl (–CH_3_) and methylene (–CH_2_) protons of the ethyl groups in the diethyl chlorophosphate, respectively. Their presence, along with the disappearance or shifting of certain characteristic peaks of the starting monomer, supports the formation of the desired phosphorus-containing product. This spectral evidence confirms that the intended chemical modification has taken place, marking a successful progression in the synthesis pathway.

**Figure 2:**
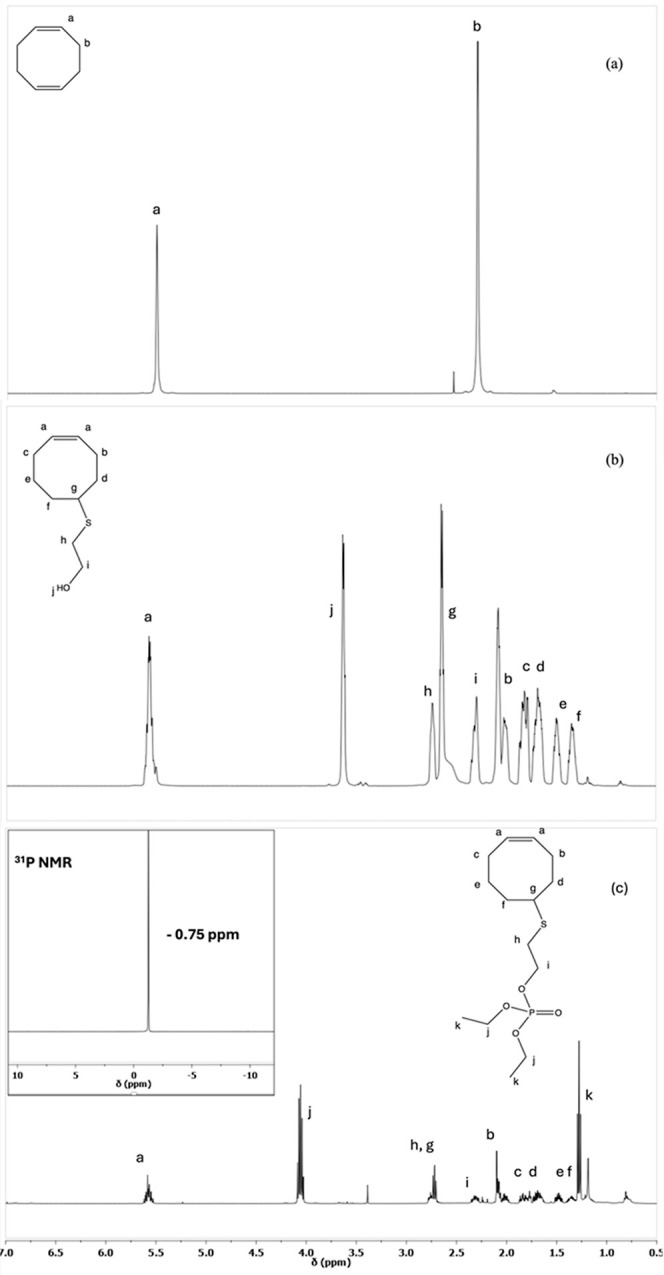
^1^H NMR spectra of (a) cyclooctadiene, (b) Compound 1, and (c) Monomer 1. Inset is the ^31^P NMR spectrum for Monomer 1.

The ^13^C NMR spectrum of cyclooctadiene (Figure 3a) displays two distinct carbon resonances, with the olefinic C–H carbons (C=C) appearing at ∼127 ppm and the aliphatic methylene (CH_2_) carbons observed at ∼27 ppm. The ^13^C NMR spectrum (Figure 3b) validated the synthesis of Compound 1. The presence of vinylic carbons from the cyclooctadiene unit was evident from the signals in the range of 124–128 ppm. The carbon atoms bonded to the thiol group were observed at 43 ppm. Additionally, the carbon adjacent to the hydroxyl (–OH) group was identified at 61 ppm. The ^13^C NMR spectrum, as shown in Figure 3c, further corroborates the successful reaction between Compound 1 and diethyl chlorophosphate. The resonance at approximately 16 ppm is attributed to the methyl carbon (–CH_3_), while the peak around 63 ppm corresponds to the methylene carbon (–CH_2_–) adjacent to the oxygen atom in the ethoxy group. The spectral data supports the formation of the phosphorus-containing compound.

**Figure 3:**
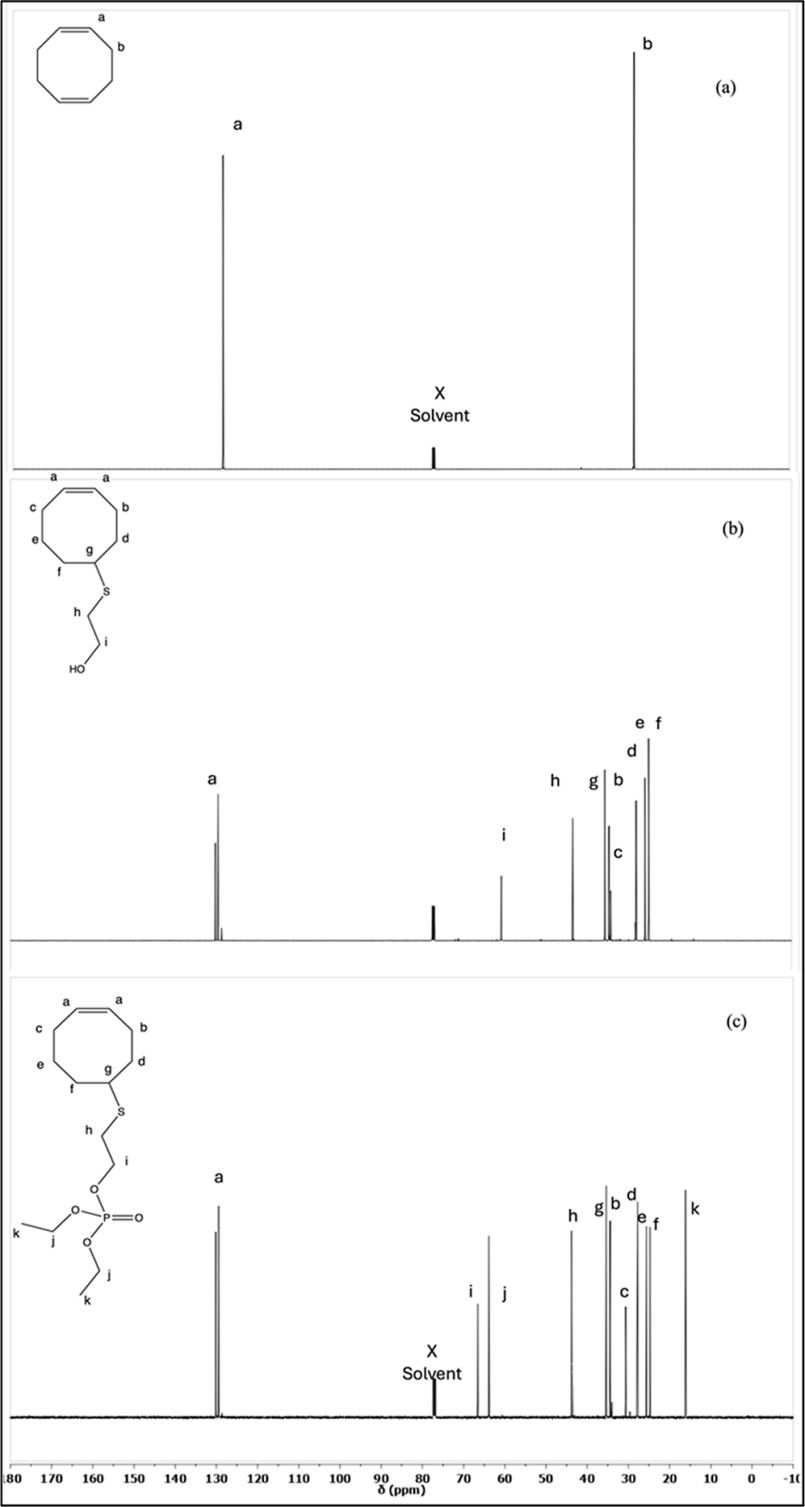
^13^C NMR spectra of (a) cyclooctadiene, (b) Compound 1, and (c) Monomer 1.

The ^31^P NMR spectrum (Figure 2c, inset) provides definitive evidence for the successful incorporation of the phosphorus moiety from diethyl chlorophosphate into Compound 1. A distinct singlet was observed for Monomer 1 at −0.75 ppm, which is characteristic of a phosphorus atom in a P­(III) environment, such as that found in phosphate esters. This chemical shift falls within the expected range for diethyl phosphate derivatives, confirming the presence of a phosphorus-containing functional group in the final product. Together with the ^1^H and ^13^C NMR data, the ^31^P NMR spectrum provides compelling structural evidence of the successful synthesis of the desired phosphorus-functionalized monomer M1.

### 3.2 Synthesis of PolyCOD_phosphonate_

ROMP is one of the most versatile and extensively investigated polymerization techniques, enabling the synthesis of polymers with precisely controlled architectures and diverse functionalities from cyclic olefin monomers. In ROMP, as well as in other olefin metathesis reactions catalyzed by Grubbs-type ruthenium complexes, vinyl ethers have long been employed as efficient chain-terminating agents. The addition of vinyl ethers rapidly reacts with the active metal carbene species, thereby quenching the catalyst and terminating the polymerization process. This quenching strategy has been a standard and reliable method for controlling ROMP and related metathesis reactions for more than three decades.^42–44^ Typical monomer 1,5-Cyclooctadiene (COD) represents a class of commonly utilized ROMP.^45^ The ROMP of COD typically affords polybutadiene with an ideal 1,4-addition microstructure.^46^ ^,^ ^47^

ROMP of the target monomer was systematically investigated using Grubbs’ second-generation (G2) and third-generation (G3) catalysts under various reaction conditions. Reaction parameters, including temperature, reaction time, and monomer-to-catalyst ratio ([M_1_]/[Cat]), were adjusted to optimize polymer formation and control molecular weight characteristics. Polymerization attempts using G2 at room temperature, 40 °C, and 60 °C for both 1 and 24 h reaction times did not yield polymeric material, suggesting that the catalyst activity was insufficient to initiate or propagate metathesis under these conditions. This lack of reactivity can be attributed to the phosphonate groups coordination to the Ru center, which limits the driving force for polymerization, combined with the modest initiation efficiency of the G2 system. Diminishing of strain in the ring also causes the inability of G2 to promote ROMP.^48^ In contrast, the third-generation Grubbs catalyst (G3) demonstrated significantly enhanced catalytic performance. While no polymerization occurred at room temperature or 40 °C, successful polymer formation was achieved at 60 °C, as observed in catalyst ratios 300:1 and 500:1, which resulted in the formation of low-molecular-weight polymers with remarkably narrow molecular weight distributions. The number-average molecular weights (*M*_n_) of ∼1.3 × 10^3^ g·mol^–1^ determined by GPC with THF as the eluent and molecular-weight calibration was carried out using polystyrene standards. The GPC molecular weight distribution curves of the two polymers, shown in Figure S3, exhibit sharp, single, and nearly overlapping peaks, characteristic of monodisperse samples. The absence of any high- or low-molecular-weight shoulders suggests uniform chain initiation and negligible chain-transfer or termination events during polymerization. These findings highlight the critical influence of catalyst generation and temperature on the success of ROMP for moderately strained monomers.^49^ The enhanced initiation rate and stability of the propagating ruthenium carbene in G3 due to the pyridine ligand substitution facilitate more efficient activation of the monomer compared to G2. Consequently, elevated temperatures (60 °C) provide the necessary thermal energy to overcome the monomer’s lower ring strain, promoting complete conversion within one hour.^50^

The ^1^H NMR spectrum (Figure 4a) provides compelling evidence for the successful polymerization of the monomer. A characteristic signal corresponding to the vinylic proton of the double bond was initially observed at 5.56 ppm in the monomer spectrum. However, upon polymerization, this signal undergoes a noticeable downfield shift to 5.32 ppm, indicating a change in the electronic environment around the vinyl groups. This shift in the chemical shift of the double-bond proton confirms that the polymerization reaction has taken place, likely through the consumption of the C=C double bond during the propagation step. The significant reduction in intensity of the original monomer peak, coupled with the appearance of the new, broadened signal at 5.32 ppm, is consistent with the formation of the polymer backbone.^51^

**Figure 4:**
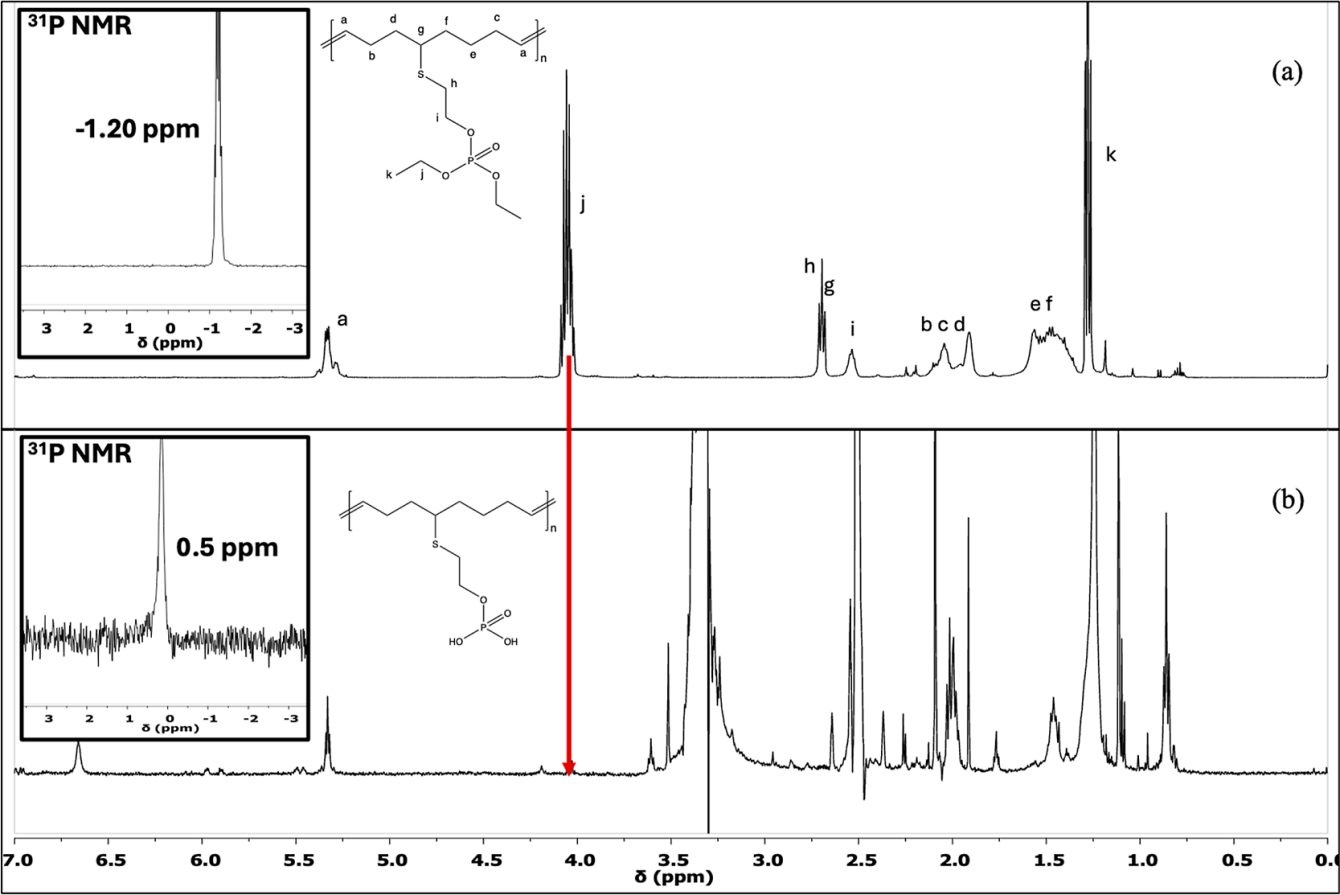
^1^H NMR spectra of (a) PolyCOD_phosphonate_ and (b) PolyCOD_phosphonic acid_ before and after cleavage of the phosphonate ester group, respectively, the spectrum of Poly COD_phosphonate_ is recorded in CDCl_3_ and that of PolyCOD_phosphonic acid_ in DMSO-*d*_6_. The insets are corresponding ^31^P NMR spectra of the respective polymers.

The ^31^P NMR spectrum (Figure 4a, inset) provides insightful evidence of the chemical environment changes around the phosphorus atom following the reaction. A notable shift in the phosphorus signal is observed, moving from −0.75 ppm in the initial compound to −1.2 ppm in the final product. This downfield shift (Δδ = 0.45 ppm) suggests a subtle but significant change in the electronic environment surrounding the phosphorus nucleus.^52^ ^,^ ^53^ The ^13^C NMR spectrum of the polymer is presented in Figure S4. Characteristic carbon signals associated with the phosphonate groups were observed at 62 ppm and 15 ppm, confirming the successful incorporation of phosphonate functionalities into the polymer backbone. Together with the ^1^H NMR results, these findings provide strong evidence for the successful chemical modification and structural evolution of the target polymer.

### 3.3 Synthesis of PolyCOD_phosphonic acid_ by Deprotection of PolyCOD_phosphonate_

For the transformation into PolyCOD_phosphonic acid_, the phosphonate ester groups in PolyCOD_phosphonate_ were cleaved using trimethylsilyl bromide (TMSiBr) followed by treatment with a methanol/dichloromethane mixture. Prior to deprotection, PolyCOD_phosphonate_ was soluble in chloroform and DCM. However, after cleavage of the phosphonate esters, the resulting polymers PolyCOD_phosphonic acid_ became insoluble in these organic solvents. The deprotection of dialkylphosphonate PolyCOD_phosphonate_ to yield PolyCOD_phosphonic acid_ proceeded nearly quantitatively, as confirmed by FTIR and NMR spectroscopies.

FTIR analysis clearly showed the disappearance of the phosphonate alkyl peaks after deprotection. A strong absorption band at 1250 cm^–1^, corresponding to the P=O stretching vibration of phosphonate esters, vanished upon cleavage (Figure 5). In addition, a broad band appeared around 3500 cm^–1^, attributed to the O–H stretching vibrations of the free phosphonic acid groups. The FTIR spectra of polymers PolyCOD_phosphonate_ also exhibited strong absorptions near 1037 cm^–1^, characteristic of P–O–C stretching vibrations. After cleavage, the disappearance of this band could not be clearly distinguished due to spectral broadening in this region.^53^

**Figure 5:**
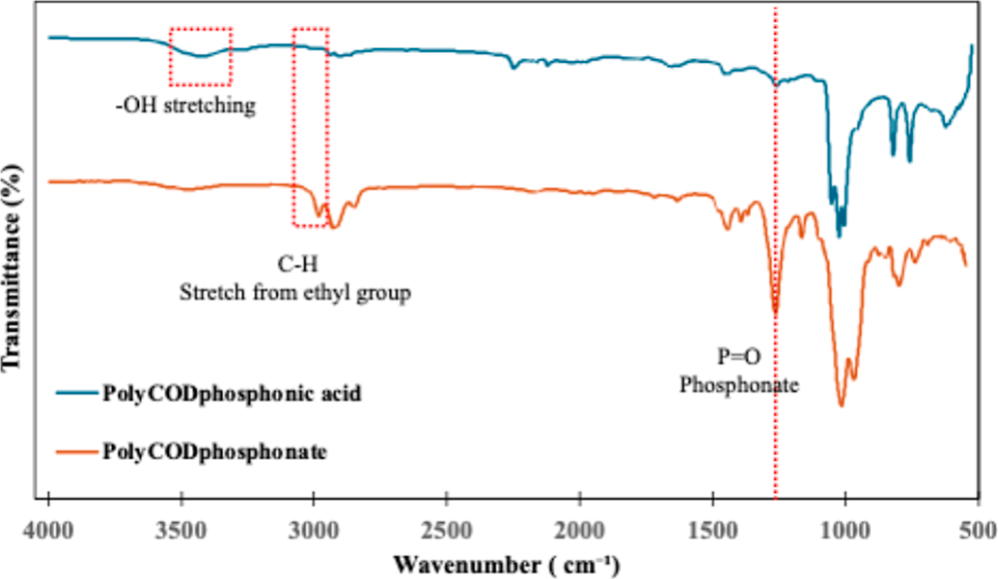
FTIR spectra for PolyCOD_phosphonic acid_ and PolyCOD_phosphonate_.

The ^1^H NMR spectrum (Figure 4b) shows the absence of the methyl proton signal, which suggests the formation of core–shell micelles, with the hydrophobic segment buried in a nonsolvated micellar core.^54^ ^,^ ^55^ The ^31^P NMR spectrum (Figure 4b, inset) exhibited a distinct chemical shift from −1.20 to 0.5 ppm, corresponding to the transformation of the phosphonate ester to the phosphonic acid. The resonance signal at −1.20 ppm was attributed to the phosphorus atom in the dialkyl phosphonate ester environment, while the upfield shift to 0.5 ppm indicated the successful cleavage of the ester groups and formation of the phosphonic acid. A comparison of the phosphonate ester and phosphonic acid functionalities is presented in Figure S5. The ^31^P NMR spectra of PolyCOD-phosphonate (red) and PolyCOD-phosphonic acid demonstrate the successful conversion of phosphonate ester groups into phosphonic acid functionalities, as evidenced by the corresponding shift in the phosphorus resonance signals.

### 3.4 Thermal Analysis and Fire Tests

#### 3.4.1 Thermogravimetric analysis (TGA)

Thermogravimetric analysis (TGA) was performed to evaluate the thermal stability of PolyCOD_phosphonate_ and its corresponding phosphonic acid derivative (Figure 6). Both polymers exhibited multistep degradation behavior, with distinct differences in their thermal stability profiles. The PolyCOD_phosphonate_ showed an initial weight loss starting around 200 °C, corresponding to the release of adsorbed moisture and low-molecular-weight volatiles. The main degradation occurred between 200 and 500 °C, associated with the decomposition of the polymer backbone, followed by the formation of a stable char residue of approximately 25% at 800 °C.^56^ In contrast, the PolyCOD_phosphonic acid_ began to degrade at a lower temperature (∼150 °C), indicating reduced thermal stability due to the evolution of polyphosphonic acid or dehydration of the acid to P_4_O_10_.^48^ PolyCOD_phosphonic acid_ showed lower char yield compared to PolyCOD_phoshonate_. The decomposition for the phosphonic acid is attributed to the presence of acidic –P­(OH)_2_ groups, which promote the polyphosphonic acid char layer. Despite this difference, both polymers retained significant char residues (15–25%), suggesting the formation of thermally stable phosphorus-containing structures at high temperatures. Overall, these results demonstrate that deprotection of the phosphonate ester to the phosphonic acid form decreases thermal stability but maintains good char-forming ability, an important characteristic for flame-retardant materials.

**Figure 6:**
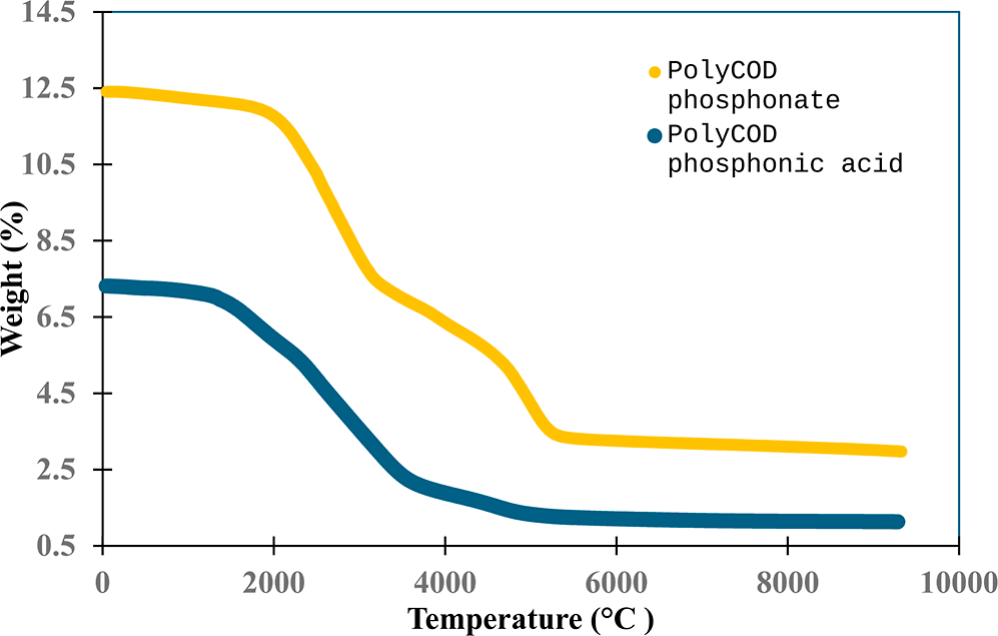
TGA spectra for PolyCOD_phosphonate_ and PolyCOD _phosphonic acid_ polymers.

#### 3.4.2 Differential Scanning Calorimetry (DSC) Analysis

The DSC measurements (Figure 7) were performed over first heating, cooling, and second heating cycles to evaluate thermal transitions and eliminate processing-related thermal history effects. During the first heating cycle, both polymers exhibit glass-transition-type behavior, confirming their predominantly amorphous nature. However, the phosphonic acid-functionalized PolyCOD displays a noticeably broader and less distinct glass transition region compared to the phosphonate ester analogue. This broadening is attributed to strong intermolecular hydrogen bonding between –PO­(OH)_2_ groups, which restricts cooperative segmental motion and leads to a distribution of relaxation times. In contrast, the phosphonate ester-functionalized PolyCOD shows a sharper and more defined *T*_g_, consistent with weaker intermolecular interactions and enhanced chain flexibility imparted by the esterified phosphorus groups. Upon cooling, neither polymer exhibits a pronounced crystallization exotherm, indicating limited crystallization ability. The absence of crystallization confirms that both polymers remain amorphous across the investigated temperature range, a desirable feature for polymer electrolyte applications. For these low-molecular-weight PolyCOD derivatives, the second heating scans did not exhibit a sharply defined glass transition, and *T*_g_ was estimated as a broad transition region rather than a single discrete value.

**Figure 7:**
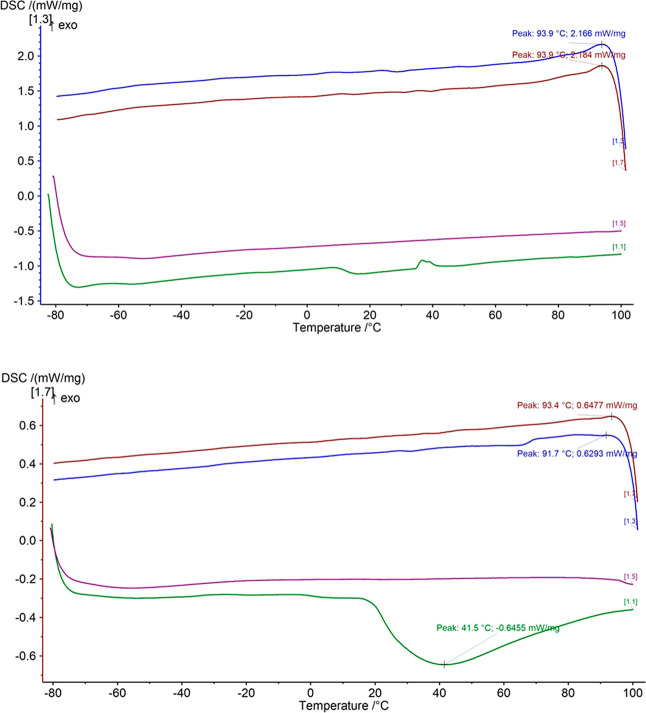
DSC thermograms of PolyCOD_phosphonate_ (upper trace) and PolyCOD_phosphonic acid_ (lower trace). Traces 1.1 and 1.3 correspond to the first heating and cooling cycles, respectively, while traces 1.5 and 1.7 represent the second heating and cooling cycles.

The PolyCOD phosphonic acid-based polymer electrolyte exhibits a significantly higher ionic conductivity (see below, 6.30 × 10^–4^ S cm^–1^) compared to its phosphonate ester analogue. This apparent discrepancy highlights that ionic conductivity in these systems is not governed mainly by segmental mobility but also by the nature and density of ion-coordinating sites.

#### 3.4.33.3.3 Microscale Combustion Calorimetry

Microscale combustion calorimetry (MCC) was employed to evaluate the combustion behavior and flame-retardant efficiency of the PolyCOD_phosphonate_ and PolyCOD_phosphonic acid_ polymer systems (Figure 8). Key combustion parameters obtained by MCC include the heat release capacity (HRC, J g^–1^ K^–1^), peak heat release rate (PHRR, W g^–1^), total heat release (THR, kJ g^–1^), and the temperature at maximum decomposition/combustion. Because MCC directly links thermal degradation chemistry to combustion energetics, it is widely used for rapid screening of flame-retardant polymers and additives to compare intrinsic fire behavior. The PolyCOD_phosphonate_ samples exhibited two distinct exothermic peaks within the temperature range of 250–420 °C, indicating a two-step degradation mechanism. The first peak, appearing around 270–300 °C, can be attributed to the initial breakdown of the polymer backbone and evolution of volatile products, while the second major peak between 380–420 °C corresponds to oxidation of the char residue or secondary degradation of intermediate species. Between these stages, a small shoulder at higher temperatures (∼600 °C) likely represents the oxidation of residual carbonaceous material. PolyCOD_phosphonate_ displayed a higher peak heat release rate and showed a lower PHRR (∼60 W g^–1^). In contrast, the PolyCOD_phosphonic acid_ presented a markedly different combustion pattern, characterized by a single dominant HRR peak at higher temperatures (approximately 500–550 °C), indicative of a more thermally stable polymeric network. The single-step degradation profile suggests that the PolyCOD_phosphonic acid_ polymers decompose through a more uniform process, producing fewer volatile intermediates than PolyCOD_phosphonate_. PolyCOD_phosphonic acid_ reached a lower PHRR (∼45 W g^–1^), reflecting improved flame-retardant behavior in the latter. The delayed decomposition and significantly lower HRR values confirm that the phosphonic acid form possesses superior thermal stability and greater condensed-phase activity. This enhancement arises from the strong hydrogen bonding and cross-linking capability of the P–OH groups, which promote char formation and reduce the release of flammable volatiles. Overall, both polymers exhibit reproducible thermal combustion profiles making it a more promising candidate for thermally stable and fire-resistant polymer materials.

**Figure 8:**
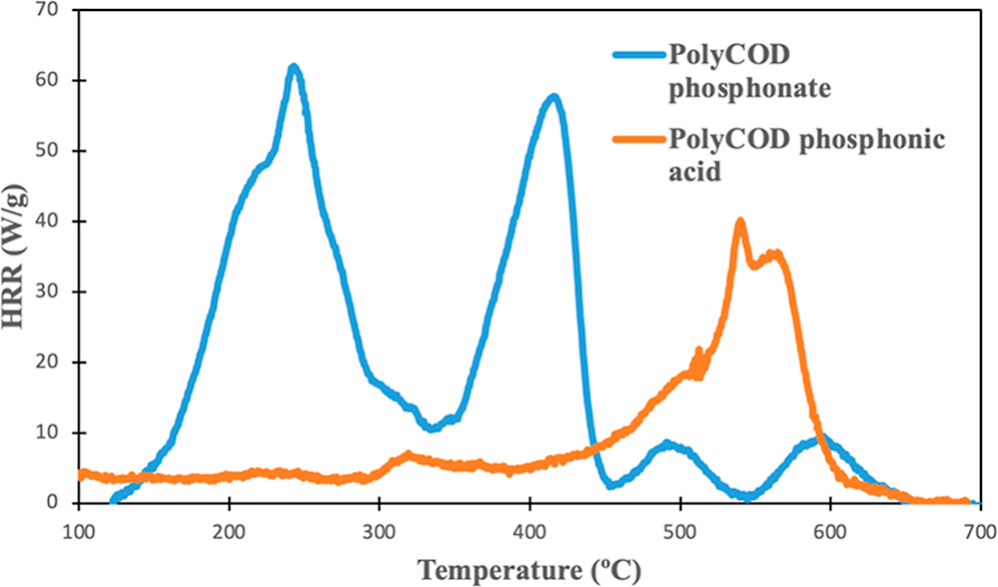
MCC spectra for Poly COD_phosphonate_ and Poly COD_phosphonic acid_.

#### 3.4.4 Vertical Burn Test

MCC does not capture macroscopic fire phenomena such as melt dripping, charring morphology evolution, or heat/mass transfer limitations and therefore is commonly complemented with larger-scale tests (e.g., LOI, UL-94, cone calorimetry) for application-level fire performance assessment. Thus, the potential flame-retardant and self-extinguishing properties of polymers were evaluated by a simple fire test. A simple vertical burn test was performed using standard laboratory filter paper treated with PolyCOD_phosphonate_ and PolyCOD_phosphonic acid_. The treatment was carried out by immersing the filter paper in a DMF solution of both polymers (0.2 g/mL) for 3 min, followed by solvent removal under vacuum. As a control, the same test was first conducted on untreated filter paper, which burned completely within 7 s, leaving no visible residue (Figure S6a). In contrast, the filter paper coated with PolyCOD_phosphonate_ and PolyCOD_phosphonic acid_ polymers exhibited an initial flare upon ignition but self-extinguished within 6 s, leaving behind a protective charred layer that resisted further combustion (Figure S6b,c). Although these results demonstrated the flame-retardant nature of PolyCOD_phosphonate_ and PolyCOD_phosphonic acid_ polymers, the noticeable initial flare and brief burn duration prompted further investigation to minimize these effects and enhance the overall fire-resistance performance. In short, the vertical burn test was performed as a preliminary qualitative screening experiment to provide an initial indication of the self-extinguishing behavior of the polymers. More quantitative and standardized flammability analyses, including LOI, UL-94, and cone calorimetry, are required for a comprehensive fire-performance evaluation.

### 3.5 Conductivity Studies

In this conductivity study, polymer electrolyte films were fabricated by incorporating 10 wt % of PolyCOD and 10 wt % of LiTFSI salt into a poly­(vinylidene fluoride) (PVDF) matrix. The preparation method varied according to the chemical functionality of the polymer system. For the PolyCOD_phosphonate_-based electrolyte, the components were dissolved in THF, whereas for the PolyCOD_phosphonic acid_-based system, DMSO was employed as the solvent due to its superior polarity and solvation capability for the acid moieties. The mixtures were magnetically stirred until a homogeneous solution was obtained, ensuring complete dissolution and uniform dispersion of the salt and polymer blend. The viscous solutions were then cast and allowed to evaporate slowly to remove the residual solvent completely. The resulting solid films were subjected to a cold-pressing process to improve film compactness and interfacial contact between the polymer chains and the dopant species. The resulting samples had a diameter of 1 cm and a thickness of 0.05 ± 0.003 mm. Each sample was positioned between two stainless-steel blocking electrodes under spring-applied pressure to ensure good interfacial contact. Electrochemical impedance spectroscopy (EIS) was employed to evaluate the impedance behavior of the polymer electrolytes. The ionic conductivity of the samples was subsequently calculated using the following equation:

1 $$\sigma =\frac{t}{R_{b}A}$$

where σ is the conductivity in S cm^–1^, *t* is the thickness in cm, *R*_b_ is the bulk resistance, and *A* is the area in cm^2^.

The Cole–Cole plots of PVDF+10%LiTFSI, PVDF+ 10%LiTFSI+10%PolyCOD_phosphonate_, and PVDF+ 10%LiTFSI+10%PolyCOD_phosphonic acid_ are depicted in Figure 9. All samples exhibit a characteristic semicircular arc in the high-frequency region, followed by a tendency toward a linear tail at lower frequencies, which is typically associated with electrode polarization effects.

**Figure 9:**
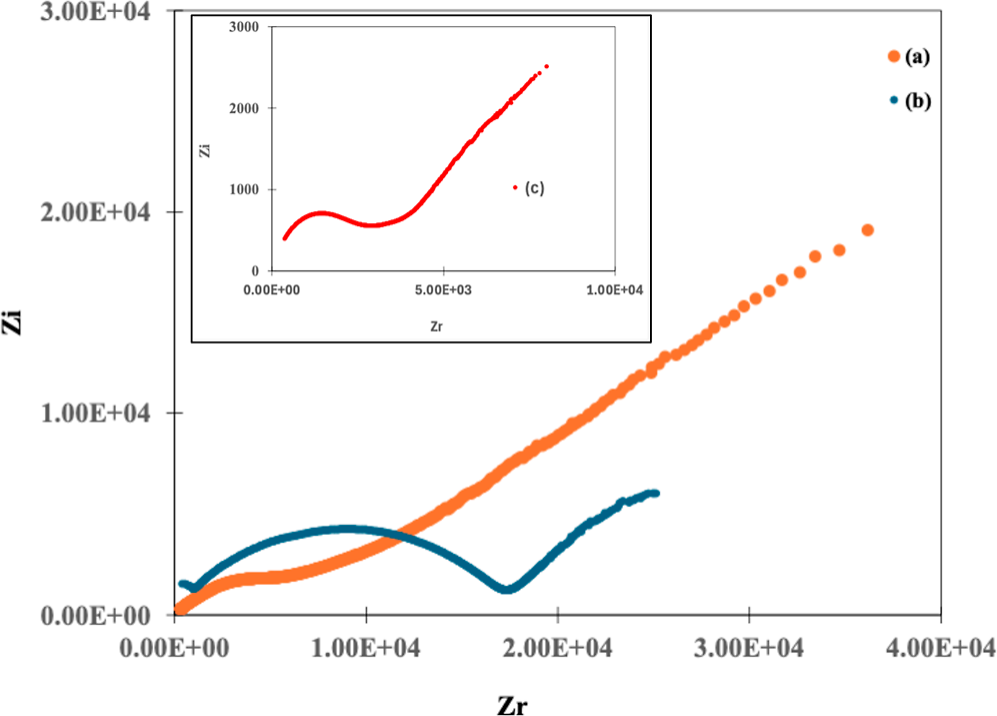
Cole–Cole plots of (a) PVDF + 10%LiTFSI, (b) PVDF + 10%LiTFSI + 10%PolyCOD_phosphonate_, and (c) PVDF + 10%LiTFSI + 10%PolyCOD_phosphonic acid_.

The Cole–Cole (Nyquist) plots of PVDF + 10% LiTFSI (a), PVDF + 10% LiTFSI + 10% phosphonate ester (b), and PVDF + 10% LiTFSI + 10% phosphonic acid (c) reveal a clear evolution in impedance behavior as a function of phosphorus-based functionalization. All samples exhibit nonideal semicircular or depressed arc features in the high-frequency region followed by a sloped tail at lower frequencies, indicating a combination of bulk ionic conduction and electrode polarization effects.^30^

The pristine PVDF + LiTFSI system (a) shows a relatively lower high-frequency intercept on the Zr axis compared with the phosphonate ester system, suggesting a lower apparent bulk resistance under the tested conditions. However, its impedance response is still relatively large, reflecting limited ion dissociation and restricted segmental mobility within the PVDF matrix.

Upon incorporation of the phosphonate ester (b), the impedance response increases, as evidenced by the larger arc and higher Zr values. This indicates an increase in bulk and/or interfacial resistance. The phosphonate ester groups, while capable of interacting with Li^+^ ions, may introduce stronger ion–polymer interactions or increased structural rigidity, which can hinder polymer chain dynamics and reduce effective ion mobility. Additionally, insufficient salt dissociation or the formation of ion aggregates may further contribute to the increased resistance observed in this system.

In contrast, the phosphonic acid-containing system (c) demonstrates a significant reduction in impedance, with markedly lower Zr and Zi values across the measured frequency range. This indicates the lowest bulk resistance and the most efficient ion transport among the three samples. The enhanced performance can be attributed to the strong polarity and proton-donating nature of the phosphonic acid groups, which facilitate more effective dissociation of LiTFSI and increase the concentration of mobile Li^+^ ions. Furthermore, the presence of –PO­(OH)_2_ functionalities promotes stronger ion–dipole interactions and may enable proton-assisted or hopping-type transport mechanisms, resulting in more continuous and efficient ion-conduction pathways.

Overall, the impedance results clearly demonstrate that the nature of the phosphorus-containing functional group plays a critical role in governing the ionic transport. While phosphonate ester incorporation leads to increased resistance, conversion to the phosphonic acid form significantly enhances the electrochemical performance. The trend in bulk resistance can be summarized as follows:

PVDF + LiTFSI + phosphonic acid < PVDF + LiTFSI < PVDF + LiTFSI + phosphonate ester.

The sample consisting of PVDF with 10 wt % LiTFSI exhibited an ionic conductivity of 1.05 × 10^–4^ S cm^–1^, which is within the typical range for PVDF-based electrolytes. The sample which included an additional 10 wt % of PolyCOD_phosphonate_ demonstrated a significantly decreased ionic conductivity of 7.0 × 10^–5^ S cm^–1^. Additionally, the PolyCOD_phosphonic acid_-based polymer electrolyte exhibited a significantly higher ionic conductivity of 6.30 × 10^–4^ S cm^–1^ compared to its phosphonate ester analogue. This enhancement can be attributed to the presence of the acidic –P­(=O)­(OH)_2_ groups, which provide additional protonic charge carriers and promote stronger ionic dissociation of the doped salt within the PVDF matrix. This finding highlights that phosphonic acid functionalization is a more effective strategy for improving ionic conductivity and reducing impedance in PVDF-based polymer electrolytes.

The cyclic voltammetry (CV) curves of the PVDF+ 10%LiTFSI+10%PolyCOD_phosphonic acid_ polymer electrolyte at scan rates of 20, 50, and 100 mV s^–1^ exhibit a quasi-rectangular shape without the presence of distinct redox peaks, indicating that the charge storage process is predominantly capacitive rather than Faradaic (Figure 10). This behavior suggests the good electrochemical stability of the electrolyte within the investigated voltage window.

**Figure 10:**
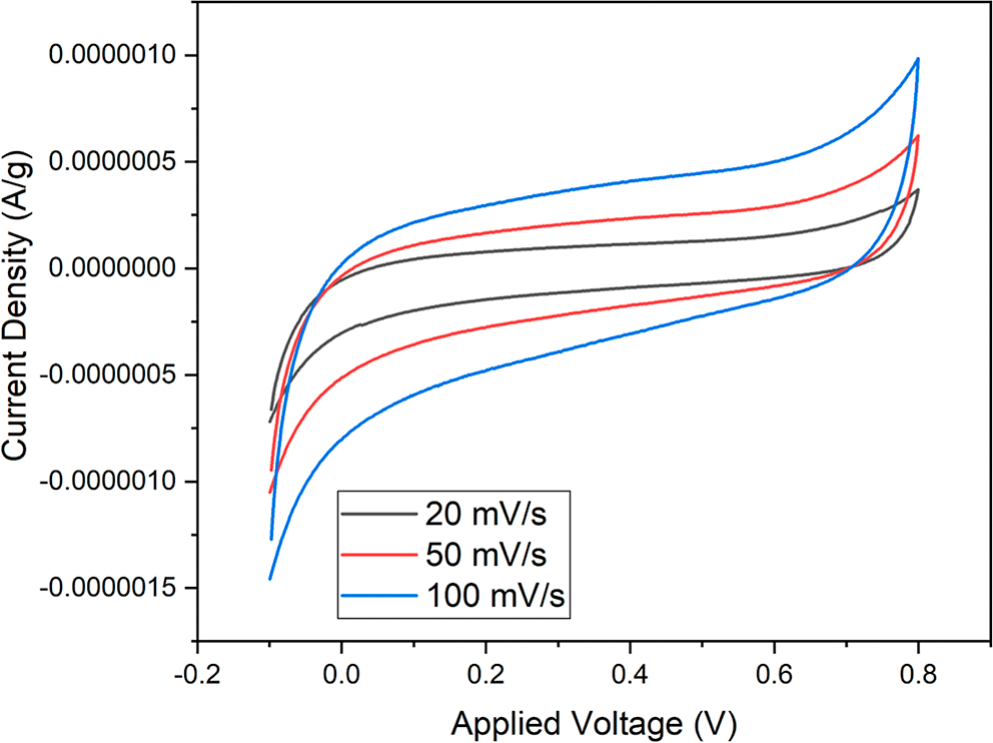
Cyclic voltammetry for PVDF + 10%LiTFSI + 10%PolyCOD_phosphonic acid_.

As the scan rate increases from 20 to 100 mV s^–1^, the current density correspondingly increases, which is a typical characteristic of capacitive systems. The enlargement of the CV loop area at higher scan rates reflects an enhanced charge accumulation and faster ion transport dynamics. The relatively symmetric shape of the curves between the forward and reverse scans further indicates good reversibility and a stable electrochemical behavior.

However, a slight distortion from an ideal rectangular shape, particularly at higher potentials and scan rates, was observed. This deviation is attributed to the internal resistance (IR drop) and polarization effects within the electrolyte system. At higher scan rates, the ions have less time to migrate and redistribute within the polymer matrix, leading to increased resistive limitations and deviations from ideal capacitive behavior.

The cyclic voltammetry profiles exhibit a quasi-rectangular shape without distinct redox peaks, indicating that the electrolyte remains electrochemically stable within the investigated potential range. This behavior confirms a wide electrochemical stability window and the absence of parasitic electrochemical reactions. The gradual increase in the current density with an increasing scan rate suggests efficient ionic transport and good reversibility. Although the Li^+^ transference number cannot be directly extracted from CV measurements, the capacitive response and lack of faradaic features imply that ionic conduction is dominant with favorable Li^+^ transport characteristics. These findings are consistent with impedance results, supporting the suitability of the electrolyte for lithium-ion battery applications.

Overall, the CV results demonstrate that the polymer electrolyte exhibits a stable electrochemical performance, good reversibility, and scan-rate-dependent capacitive characteristics. The increase in the current response with scan rate confirms efficient ion transport, although minor polarization effects become more pronounced at higher scan rates.

## 4 Conclusion

In this study, we successfully synthesized and characterized phosphorus containing polymers, PolyCOD_phosphonate_ and PolyCOD_phosphonic acid_, as solid polymer electrolytes for safer and high-performance lithium-ion batteries (LIBs). The polymer was prepared via thiol–ene click chemistry followed by phosphonate functionalization and ring-opening metathesis polymerization (ROMP) using Grubbs’ third-generation catalyst. Blending PolyCOD_phosphonate_ and PolyCOD_phosphonic acid_ with polyvinylidene difluoride (PVDF) and lithium bis­(trifluoromethanesulfonyl)­imide (LiTFSI) yielded a composite electrolyte with excellent thermal stability (>300 °C) and enhanced ionic conductivity (6.30 × 10^–4^ S cm^–1^) at ambient temperature. The strong solvation ability of the phosphonate moieties facilitated efficient lithium-ion transport, while their inherent flame-retardant properties improved safety. These findings demonstrate that phosphonate-functionalized polycyclooctene is a promising candidate for next-generation solid-state LIBs, combining high ionic conductivity, thermal resilience, and enhanced safety key requirements for advanced energy storage systems. Future work will include Li^+^ transference number determination, extended electrochemical stability analysis, and coin cell testing to evaluate cycling performance and interfacial stability, alongside further optimization of polymer structure for enhanced ionic conductivity.

## Supplementary Material



## Notes

Experimental procedures; materials and instrumentation; synthesis of Compound 1, Monomer 1, PolyCOD_phosphate_, and PolyCOD_phosphonic acid_; preparation of solid polymer electrolyte membranes; burn test methodology; GPC analysis; ^13^C and ^31^P NMR spectra; TLC analysis; and vertical burn test photographs (PDF)

## References

[1] Megahed S., Scrosati B. (1994). Lithium-Ion Rechargeable Batteries. J. Power Sources.

[2] Ram Prasanth, S. ; Prasannavenkadesan, V. ; Katiyar, V. ; Achalkumar, A. S. Polymer Electrolytes: Evolution, Challenges, and Future Directions for Lithium-Ion Batteries. Royal Society of Chemistry 2024, 3(3).

[3] Teece D. J. (2018). Tesla and
the Reshaping of the Auto Industry. Manag. Organ.
Rev..

[4] Schulze A., MacDuffie J. P., Täube F. A. (2015). Introduction: Knowledge Generation
and Innovation Diffusion in the Global Automotive Industry—Change
and Stability during Turbulent Times. Ind. Corp.
Chang..

[5] Covarrubias V, A. ; Ramírez Pérez, S. M. , Eds. New Frontiers of the Automobile Industry: Exploring Geographies, Technology, and Institutional Challenges; Palgrave Macmillan: Cham, Switzerland, 2020. 10.1007/978-3-030-18881-8

[6] Ferràs-Hernández X., Tarrats-Pons E., Arimany-Serrat N. (2017). Disruption in the Automotive Industry:
A Cambrian Moment. Bus. Horiz..

[7] Mordue G. (2020). Shifting Patterns
in the Application of Industrial Policy. Int.
J. Automot. Technol. Manag..

[8] Yu X., Manthiram A. (2021). A Review of
Composite Polymer-Ceramic Electrolytes
for Lithium Batteries. Energy Storage Mater..

[9] Kim T., Song W., Son D.-Y., Ono L. K., Qi Y. (2019). Lithium-Ion
Batteries: Outlook on Present, Future, and Hybridized Technologies. J. Mater. Chem. A.

[10] Goodenough J. B., Park K. S. (2013). The Li-Ion
Rechargeable Battery: A Perspective. J. Am.
Chem. Soc..

[11] Kim, H.-J. , Krishna, T. N. V. , Zeb, K. , Rajangam, V. , Gopi, C. V. V. M. , Sambasivam, S. , Raghavendra, K. V. G. , Obaidat, I. M. A Comprehensive Review of Li-Ion Battery Materials and Their Recycling Techniques. Electronics 2020, 9 (7), 1161. 10.3390/electronics9071161

[12] Bachman J. C. (2016). Inorganic Solid-State Electrolytes for Lithium Batteries: Mechanisms
and Properties Governing Ion Conduction. Chem.
Rev..

[13] Nitta N., Wu F., Lee J. T., Yushin G. (2015). Li-Ion Battery Materials: Present
and Future. Mater. Today.

[14] Manthiram A., Yu X., Wang S. (2017). Lithium Battery Chemistries
Enabled by Solid-State
Electrolytes. Nat. Rev. Mater..

[15] Misenan M. S. M., Isa M. I. N., Khiar A. S. A. (2018). Electrical and
Structural Studies
of Polymer Electrolyte Based on Chitosan/Methyl Cellulose Blend Doped
with BMIMTFSI. Mater. Res. Express.

[16] Misenan M. S. M., Khiar A. S. A., Eren T. (2022). Polyurethane-Based
Polymer Electrolyte
for Lithium Ion Batteries: A Review. Polym.
Int..

[17] Misenan M. S. M., Hempelmann R., Gallei M., Eren T. (2023). Phosphonium-Based Polyelectrolytes:
Preparation, Properties, and Usage in Lithium-Ion Batteries. Polymers.

[18] Misenan, M. S. M. ; Ali, E. S. ; Khiar, A. S. A. Conductivity, Dielectric and Modulus Study of Chitosan–Methyl Cellulose–BMIMTFSI Polymer Electrolyte Doped with Cellulose Nano Crystal. AIP Conference Proceedings 2018, 1972 (1), 030010. 10.1063/1.5041231

[19] Shaffie, A. H. ; Misenan, M. S. M. ; Isa, M. I. N. ; Khiar, A. S. A. Effect of Ionic Liquid BMIMNO_3_ to Chitosan-Starch Blend Biopolymer Electrolyte System. Solid State Phenom. 2019, 290, 177−182. 10.4028/www.scientific.net/SSP.290.177

[20] Misenan, M. S. M. ; Shaffie, A. H. ; Khiar, A. S. A. Effect of BMITFSI to the Electrical Properties of Chitosan/Methylcellulose Based Polymer Electrolyte. AIP Conference Proceedings 2018, 1972 (1), 030001. 10.1063/1.5041222

[21] Misenan M. S. M., Farabi M. S. A., Akhlisah Z. N., Khiar A. S. A. (2025). Enhancing Polymer
Electrolytes with Carbon Nanotube Fillers: A Promising Frontier. Next Mater..

[22] Misenan, M. S. M. ; Khiar, A. S. A. Conduction Mechanism of Chitosan/Methylcellulose/1-Butyl-3-Methyl Imidazolium Bis­(Trifluoromethylsulfonyl) Imide (BMIMTFSI) Biopolymer Electrolyte Doped with Ammonium Triflate. Malaysian J. Chem. 2020, 22(4), 1−13. 10.55373/mjchem.v22i4.781

[23] Misenan M. S. M., Khiar A. S. A. (2020). Structural Studies and Ionic Transport Properties of
Solid Biopolymer Electrolytes Based on Chitosan/Methyl Cellulose Blend
Doped with BMIMTFSI. Solid State Phenom..

[24] Misenan, M. S. B. ; Farabi, M. S. A. ; Akhlisah, Z. N. ; Azlisham, N. A. F. Preparation, Characterization and Application of Nanocellulose from Tunicate for Electronic Applications. In Polymer Composites Derived from Animal Sources; Woodhead Publishing, 2024; pp 295–319.

[25] Janek J., Zeier W. G. (2016). A Solid Future for
Battery Development. Nat. Energy.

[26] Wakefield
IV H., Kevlishvili I., Wentz K. E., Yao Y., Kouznetsova T. B., Melvin S. J., Ambrosius E. G., Herzog-Arbeitman A., Siegler M. A., Johnson J. A., Craig S. L., Kulik H. J., Klausen R. S. (2023). Synthesis and Ring-Opening Metathesis Polymerization
of a Strained trans-Silacycloheptene and Single-Molecule Mechanics
of Its Polymer. J. Am. Chem. Soc..

[27] He Y. (2019). Preparation of Polymer
Electrolyte Membranes with Continuous PEG
Channel via the Fusion of Self-Assembled Polycyclooctene-graft-Polyethylene
Glycol Copolymer Micelles. J. Membr. Sci..

[28] Dorigato A., Pegoretti A. (2018). Evaluation
of the Shape Memory Behavior of a Poly­(cyclooctene)-Based
Nanocomposite Device. Polym. Eng. Sci..

[29] Harakawa H., Okabe M., Nomura K. (2020). The Synthesis of Cyclic Olefin Copolymers
(COCs) by Ethylene Copolymerisations with Cyclooctene, Cycloheptene,
and with Tricyclo­[6.2.1.0­(2,7)]­Undeca-4-Ene: The Effects of Cyclic
Monomer Structures on Thermal Properties. Polym.
Chem..

[30] Weichelt F., Lenz S., Tiede S., Reinhardt I., Frerich B., Buchmeiser M. R. (2010). ROMP-Derived
Cyclooctene-Based Monolithic
Polymeric Materials Reinforced with Inorganic Nanoparticles for Applications
in Tissue Engineering. Beilstein J. Org. Chem..

[31] Chen Y. (2021). A Flame Retarded Polymer-Based Composite Solid Electrolyte Improved
by Natural Polysaccharides. Compos. Commun..

[32] Zhu G. R., Zhang Q., Liu Q. S., Bai Q. Y., Quan Y. Z., Gao Y., Wu G., Wang Y. Z. (2023). Non-Flammable
Solvent-Free Liquid
Polymer Electrolyte for Lithium Metal Batteries. Nat. Commun..

[33] Stevens R., van Es D. S., Bezemer R., Kranenbarg A. (2006). The Structure–Activity
Relationship of Fire Retardant Phosphorus Compounds in Wood. Polym. Degrad. Stab..

[34] Wendels S., Chavez T., Bonnet M., Salmeia K. A., Gaan S. (2017). Recent Developments
in Organophosphorus Flame Retardants Containing P–C Bond and
Their Applications. Materials.

[35] Ge J. (2019). Phosphonate-Functionalized Ionic Liquid: A Novel Electrolyte Additive
for Enhanced Cyclic Stability and Rate Capability of LiCoO2 Cathode
at High Voltage. ChemistrySelect.

[36] Kim J., Hyun J., Kim S., Park W. H., Yu S. (2025). Phosphorus-Based
Flame-Retardant Electrolytes for Lithium Batteries. Adv. Energy Mater..

[37] Cao X. (2019). Nonflammable Electrolytes
for Lithium Ion Batteries Enabled by Ultraconformal
Passivation Interphases. ACS Energy Lett..

[38] Liu G., Cao Z., Zhou L., Zhang J., Sun Q., Hwang J., Cavallo L., Wang L., Sun Y., Ming J. (2020). Additives
Engineered Nonflammable Electrolyte for Safer Potassium Ion Batteries. Adv. Funct. Mater..

[39] He Z., Wang G., Wang C., Guo L., Wei R., Song G., Pan D., Das R., Naik N., Hu Z. (2021). Overview of Anion Exchange
Membranes Based on Ring
Opening Metathesis Polymerization (ROMP). Polym.
Rev..

[40] Misenan M. S. M., Süer N. C., Canli N. Y., Khiar A. S. A., Eren T. (2024). Synthesis of Oxanorbornene-Based
Phosphonium Polymeric
Ionic Liquids (PILs) and Investigation of Their Electrical Properties. Mater. Adv..

[41] Wu Z., Nguyen S. T., Grubbs R. H., Ziller J. W. (1995). Reactions of Ruthenium
Carbenes of the Type (PPh3)­2­(X)­2Ru:CH–CH:CPh2 (X = Cl and CF3COO)
with Strained Acyclic Olefins and Functionalized Olefins. J. Am. Chem. Soc..

[42] Hillmyer M. A., Nguyen S. T., Grubbs R. H. (1997). Utility of a Ruthenium Metathesis
Catalyst for the Preparation of End-Functionalized Polybutadiene. Macromolecules.

[43] Ogba O. M., Warner N. C., O’Leary D. J., Grubbs R. H. (2018). Recent Advances
in Ruthenium-Based Olefin Metathesis. Chem.
Soc. Rev..

[44] Martinez H., Ren N., Matta M. E., Hillmyer M. A. (2014). Ring-Opening Metathesis Polymerization
of 8-Membered Cyclic Olefins. Polym. Chem..

[45] Louie J., Grubbs R. H. (2002). Metathesis of Electron-Rich Olefins: Structure and
Reactivity of Electron-Rich Carbene Complexes. Organometallics.

[46] Starvaggi F. A., Suslick B. A., Xia Y. (2024). Ring Opening
Metathesis Polymerization
of Cyclooctadiene and Cyclooctene with Dihydrofuran: Influence of
Ru Fischer Carbene. ACS Macro Lett..

[47] Stewart K. A., Ivannikava D. A., Massouh C. M., Lessard J. J. (2025). Molecular
Weight
Control in Frontal Ring-Opening Metathesis Polymerization. Angew. Chem., Int. Ed..

[48] Neary W. J., Kennemur J. G. (2017). Variable Temperature
ROMP: Leveraging Low Ring Strain
Thermodynamics to Achieve Well-Defined Polypentenamers. Macromolecules.

[49] Bielawski C. W., Grubbs R. H. (2001). Increasing the Initiation Efficiency of Ruthenium-Based
Ring-Opening Metathesis Initiators: Effect of Excess Phosphine. J. Am. Chem. Soc..

[50] Monaco G., Zambelli A. (2005). Simple Trends in NMR
Spectra of Vinyl Polymers: The
^1H NMR Spectrum of Poly­(propylene). Macromol.
Chem. Phys..

[51] Transue W. J., Dai Y., Riu M. L. Y., Wu G., Cummins C. C. (2021). ^31P NMR Chemical
Shift Tensors: Windows into Ruthenium Phosphinidene Complex Electronic
Structures. Inorg. Chem..

[52] Liu Y., Gao L., Yu Z. (2025). Quantitative ^31P NMR Spectroscopy:
Principles,
Methodologies, and Applications in Phosphorus-Containing Compound
Analysis. Appl. Sci..

[53] Eren T. D. (2008). Phosphonic
Acid-Based Amphiphilic Diblock Copolymers Derived from ROMP. J. Polym. Sci. Part A Polym. Chem..

[54] Liu S., Weaver J. V. M., Tang Y., Billingham N. C., Armes S. P., Tribe K. (2002). Synthesis of Shell Cross-Linked Micelles
with pH-Responsive Cores Using ABC Triblock Copolymers. Macromolecules.

[55] Kahraman G., Wang D. Y., von Irmer J., Gallei M., Hey-Hawkins E., Eren T. (2019). Synthesis and Characterization
of Phosphorus- and Carborane-Containing
Polyoxanorbornene Block Copolymers. Polymers.

[56] Turgut G., Işıksel E., Kahraman G., Eren T., Özkoç G. (2018). Synthesis
of Phosphorus- and Phenyl-Based ROMP Polymers and Investigation of
Their Effects on the Thermomechanical and Flammability Properties
of a Polypropylene–IFR System. J. Appl.
Polym. Sci..

