# Supplementary material for: Phosphonate- and Phosphonic Acid-Functionalized Polycyclooctenes Enabling High Ionic Conductivity and Intrinsic Flame Retardancy in Solid-State Lithium-Ion Batteries
Source: ACS Omega. 2026 Jun 8;11(24):35728–39. doi: 10.1021/acsomega.6c02084 (PMC13294920; doi:10.1021/acsomega.6c02084)
Supplement: Supplementary file 1 [file ao6c02084_si_001.pdf]

Supporting Information

Phosphonate and Phosphonic Acid Functionalized Polycyclooctenes Enabling High Ionic Conductivity and Intrinsic Flame Retardancy in Solid-State Lithium-Ion Batteries

M. S. M. Misenan[1], Aze Ilgın Gündoğdu[1], Maheen Rahim[1], Aysel Kantürk Figen[2,3] and Tarık Eren[1]*

[1]Department of Chemistry, School of Arts and Science, Yildiz Technical University, Istanbul Turkiye.

[2]Department of Chemical Engineering, Faculty of Chemistry and Metallurgy, Yıldız Technical University, İstanbul, 34220, Türkiye

[3]BATLAB Research Center, Clean Energy Technologies Institute, Yıldız Technical University, Istanbul, Türkiye

1.0 Experimental

1.1 Materials

2-ethanol mercapto, 1,5 cyclooctadiane, diethylchlorophosphate, triethylamine, trimethylsilane bromo, polyvinyl fluoride (PVdF) and lithium bis(trifluoromethane sulfonyl) imide (LiTFSI) (99%) was purchased from Aldrich. Grubbs second-generation catalyst was supplied by Aldrich. After that Grubbs catalyst 3rd generation [$(H_2$-Imes)(3-Br-py)$_2$-$(Cl)_2$Ru=CHPh] was prepared freshly from it in according to the previous literature [1]. Acetone, ethyl vinyl ether, acetic anhydride, methanol, DMSO, pentane, chloroform, hexane, ethyl acetate, diethyl ether, petroleum ether, dichloromethane (DCM) were also obtained from Aldrich as received.

1.2 Instrumentation

A PerkinElmer Fourier transform infrared (FTIR) spectrophotometer (Model FTIR – Spectrum 400) equipped with attenuated total reflection (ATR) was used to conduct the FTIR analysis. The infrared beam was passed through with a resolution of 4cm^{-1} at a frequency in the range of 4000 to 400 cm^{-1}.

^{1}H NMR (500 MHz) and ^{13}C NMR (75 MHz) spectra were collected in $CDCl_3$ on a Bruker Avance III 500 MHz spectrometer. A Varian Mercury VX 400 MHz BB spectrometer was used to obtain ^{31}P NMR spectra.

Gel permeation chromatography (GPC) measurements were performed using an Agilent system equipped with a refractive index detector (RID). The analyses were carried out at 23 °C using tetrahydrofuran (THF) as the eluent at a flow rate of 0.30 mL min^{-1}. The sample concentration was 1.0 g L^{-1}, and an injection volume of 20 μL was used. Molecular-weight calibration was carried out using polystyrene standards.

A DSC instrument (HP DSC3, Mettler-Toledo Co.) was used to examine the standard polymer samples. The polymer content in the sample pan was 6.5 ± 0.5 mg. The samples were heated from -80°C to 100°C at a heating rate of 10 °C min^{-1} under nitrogen gas flow.

Thermogravimetric analysis (TGA) was carried out on a PerkinElmer Diamond TG/DTA instrument (Seiko Instruments SII, Exstar 6300 TG/DTA). Approximately 5–10 mg of the sample was placed in an alumina crucible and heated from 26 to 800 °C at a constant rate of 10 °C min^{-1} under a nitrogen atmosphere.

Microscale combustion calorimetry (MCC) was conducted on a Fire Testing Technology (FTT) MCC-2 instrument. Approximately 5.1 mg of each sample was placed in the sample holder and heated at a constant rate of 1 °C s^{-1} under a nitrogen flow of 80 mL min^{-1}. The volatile thermal degradation products were subsequently mixed with oxygen (20 mL min^{-1}) in the combustor, maintained at 900 °C. The heat release rate (HRR) and total heat release (THR) were recorded as functions of temperature to evaluate the flammability characteristics of the materials.

The AC conductivity properties of the polymer electrolytes were measured using a Hioki IM3536 LCR meter at various temperatures from 30 °C to 100 °C within 10 °C intervals. 10 mm diameter pressed pellets or dry cast membrane samples were sandwiched between gold plated blocking electrodes on a Novocontrol BDS 1200 sample holder, and the conductivities were measured in the frequency range from 4 Hz to 8 MHz; the absolute value of AC. The cold press technique was used to press all the polymeric ionic liquid electrolyte films. The samples were 1 cm in diameter with a thickness of 0.05 ± 0.003 μm. Under spring pressure, the samples were placed between two stainless steel blocking electrodes. The impedance of the polymer electrolyte was determined using the complicated impedance approach. The conductivity of the samples was determined using the following equation:

$$\sigma = \frac{t}{R_b A} \tag{1}$$

where σ = conductivity in S cm^{-1}, t is thickness in cm, R_b = bulk resistance and A is area in cm^2.

The electrochemical measurements were carried out in a 100 mL Schott bottle using a conventional three-electrode configuration. An Ag/AgCl electrode (3 M KCl) served as the reference electrode (RE), while a high-purity graphite rod (length: 152 mm, diameter: 6.15 mm; 99.9995% C, Alfa Aesar) was used as the counter electrode (CE). The working electrode (WE) was either a graphite rod of identical specifications or a glassy carbon electrode

(diameter: 6.096 mm, type 1, 0.44 g cm^{-1}, Alfa Aesar), which were employed separately. All electrodes were connected to a potentiostat for electrochemical analysis.

A 0.5 M sulfuric acid solution was used as the supporting electrolyte. Cyclic voltammetry (CV) measurements were performed to investigate the effect of scan rate on the electrochemical behavior, with scan rates ranging from 5 to 100 mV s^{-1}. Additionally, CV measurements were conducted within a potential window of −0.2 to 0.8 V at selected scan rates of 20, 50, and 100 mV s^{-1}.

1.3 Synthesis of cyclooct-4-en-1-ylthio methanol (Compound 1).

1 mL (0.0143 mol) of mercapto ethanol was mixed with 6 mL (0.0447 mol) of cyclooctadiene in the quartz tube. 0.01 g (4.46×10^{-5} mol) of irgacure 2959 was added into the solution. The reaction occurred in photoreactor (equipped with a 365 nm, 100 W lamp) for 6 hours. After the reaction, the sample was undergone purification via column chromatography (3:2, v/v Hexane:ethyl acetate) and monitored by thin layer chromatography (**Figure S1)**. All the solvent was evaporated by rotavapor. The colourless liquid product was obtained (70% yield).

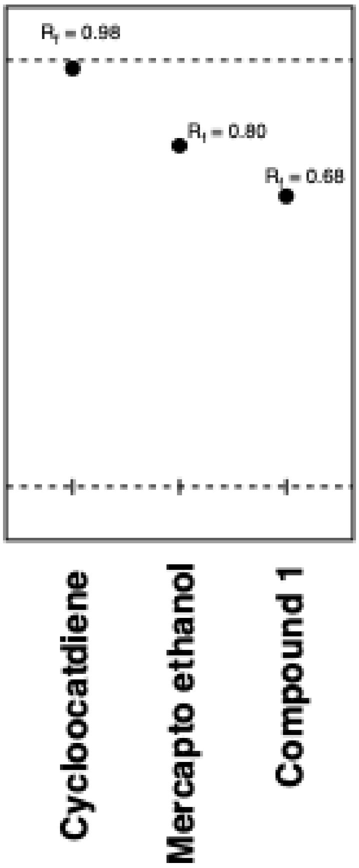

Figure S1. TLC analysis of Compound 1. (3:2, v/v hexane: ethyl acetate).

1.4 Synthesis of diethoxy[(cyclooct-2-en-1-yl)sulfanylmethoxy]methylphosphane (Monomer 1)

2 g of Compound 1 monomer was dissolved in 20 mL THF in round bottom flask. 2 ml (0.0143 mol) of triethyl amine was added into the solution. The round bottom flask was placed in the ice bath. 1.6 mL (0.0115 mol) of diethylcholoro phosphate was slowly added into the solution. The reaction occurred in room temperature for 2 days. The solution was filtered by filter paper and THF was removed. Then, 40 mL of DCM was added into the round bottom flask. Three times 20 mL of distilled water was added to sample and the sample was undergoes extraction by separation funnel. DCM phase was collected and the solvent was removed using a rotary evaporator.

The sample was undergone purification via column chromatography (3:2 Hexane:ethyl acetate) and monitored by thin layer chromatography (**Figure S2**). All the solvent was evaporated by rotary evaporator and dried in the vacuum oven for 1 days. The yellowish liquid was obtained (81% yield)

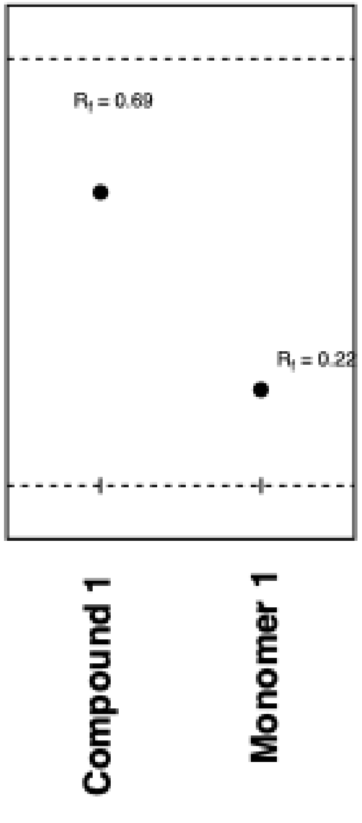

Figure S2 TLC analysis of monomer (3:2 Hexane:ethyl acetate).

1.5 Synthesis of PolyCOD$_{phosphate}$.

In a typical example polymerization procedure, Grubbs 3rd generation catalyst was dissolved in 0.5 mL of THF , and added all at once to the strongly swirling monomer solution (in 1 mL of THF). The reaction mixture was agitated for 1 h at 60°C. A further injection of 0.5 mL of 30% ethyl vinyl ether (in dichloromethane) stopped the process. Polymers were precipitated and washed with either tetrahydrofuran or diethyl ether and dried under nitrogen. Then the polymers undergo anion exchange procedure. Polymers were dissolved in deuterated $CDCl_3$ for NMR characterization.

1.6 Synthesis of PolyCOD phosphonic acid by deprotaction of PolyCOD phosphate.

In accordance with the procedure reported in the literature [2], trimethylsilyl bromide (1.5 mL, 11.5 mmol) was slowly added to a solution of compound PolyCOD phosphate (0.1 g, 0.074 mol) in 8 mL of dry dichloromethane. The reaction mixture was stirred under reflux for 4 h. The excess trimethylsilyl bromide and solvent were then removed under reduced pressure. Subsequently, 20 mL of a methanol/CH_2Cl_2 mixture (3:1 v/v) was added, and the mixture was stirred for 48 h at room temperature. After evaporation of the solvent, the polymer was purified by precipitation in excess methanol, residue was washed several times with diethyl ether and dried under vacuum. affording a orange wish solid polymer in quantitative yield (0.13 g, 99% yield).

1.7 Preparation of Solid Polymer Electrolyte Samples

Solid polymer electrolyte membranes were prepared using poly(vinylidene fluoride) (PVDF) as the host matrix. Two formulations were developed:

- **Sample 1:** PVDF with 10 wt% lithium bis(trifluoromethanesulfonyl)imide (LiTFSI) and PolyCOD$_{phosphonate}$
- **Sample 2:** PVDF with 10 wt% LiTFSI and 10 wt% PolyCOD$_{phosphonic\ acid}$

Accurately weighed amounts of PVDF, LiTFSI, and PolyCOD phosphonate were dissolved in THF under continuous magnetic stirring at room temperature. The mixtures were stirred until clear and homogeneous solutions were obtained, ensuring complete dissolution of all components. The resulting polymer solutions were cast into clean Petri dishes and allowed to dry under a fume hood at ambient temperature for approximately 12–16 hours to ensure complete removal of the THF. For PolyCOD$_{phosphonic\ acid,}$ DMSO was used as the solvent instead

of THF, and drying was carried out at 160 °C to ensure complete removal of residual solvent The dried films were then cold-pressed using a manual hydraulic press at room temperature. Uniform pressure was applied to produce compact and consistent membranes in pellet form. The thickness of the final pellets was measured using a micrometer, and all samples were prepared with an average thickness of 0.35 mm, suitable for subsequent characterization.

1.8 Preparation of Samples for Qualitative Burn Tests

Pre-cut filter paper samples (3 × 7 cm) were immersed in a DMSO solution of polymer 10 or 15 (0.2 g/mL) for 3 minutes. The treated samples were then removed from the solution and placed under vacuum for 1 day to ensure complete removal of excess solvent. The flame retardancy of the coated filter paper samples was qualitatively evaluated using an in-lab direct vertical flame test. Each sample was securely fixed in a stainless-steel clamp and positioned approximately 10 cm from the tip of a propane torch. Upon ignition, the samples were exposed to the flame, and the time required for the flame to burn through each sample was recorded. The flame propagation process was documented using a video camera, with still images captured at 0 seconds, 8 seconds,

2.0 GPC Analysis

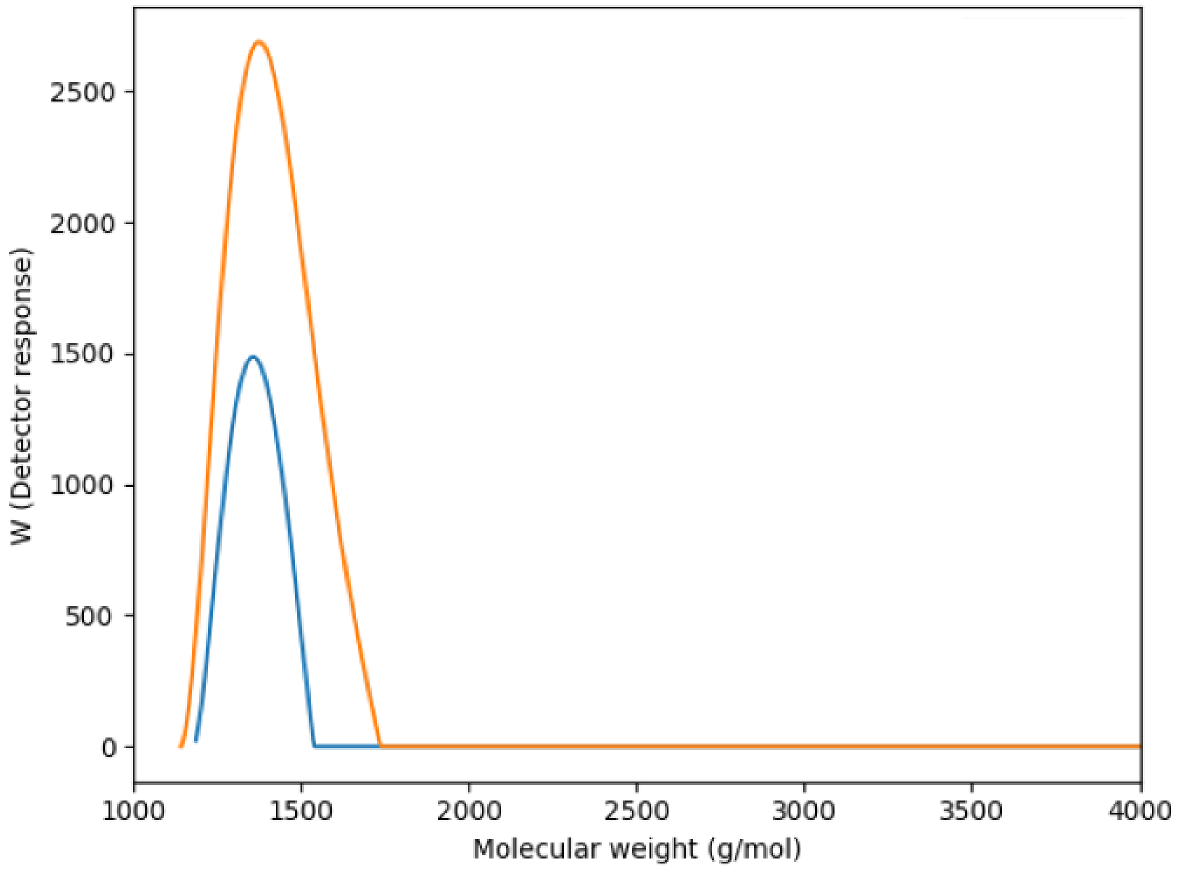

Figure S3. GPC molecular weight distribution curves of polymers obtained from the ROMP of the target monomer using the G3 catalyst at 60 °C. The samples 11th entry ([M_1]:[Cat] = 300:1) and 12th entry ([M_1]:[Cat] = 500:1) plotted in orange and blue respectively show narrow, nearly monodisperse profiles, consistent with controlled polymerization behaviour.

3.0 NMR Analysis

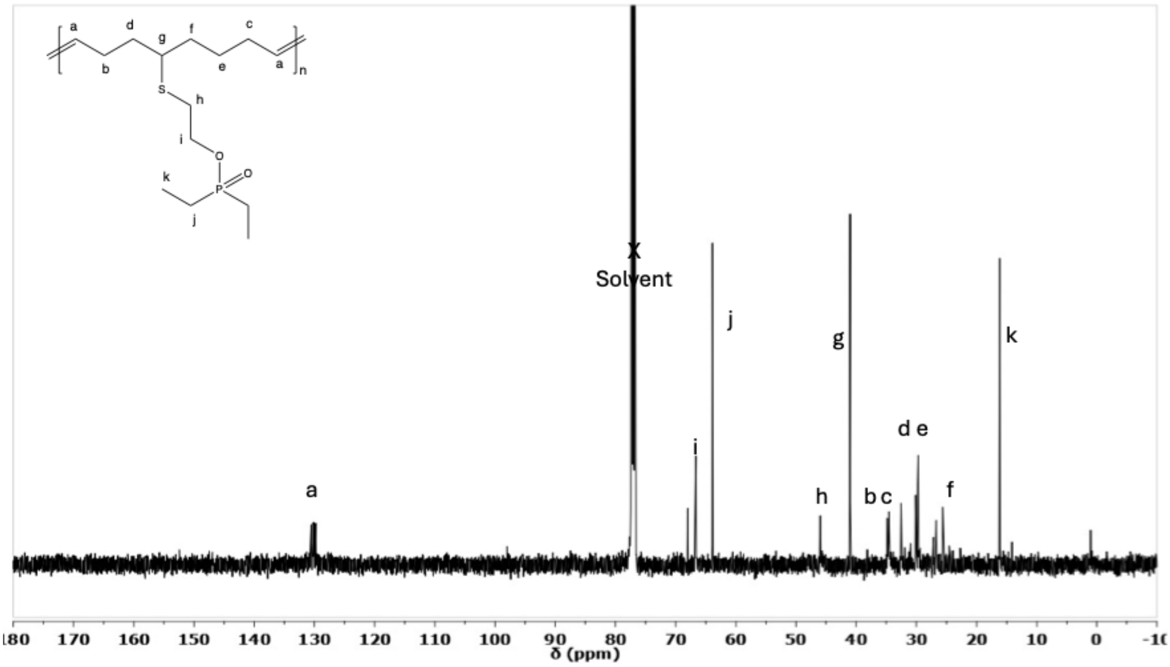

Figure S4. ^{13}C NMR spectrum of $PolyCOD_{phosphonate}$.

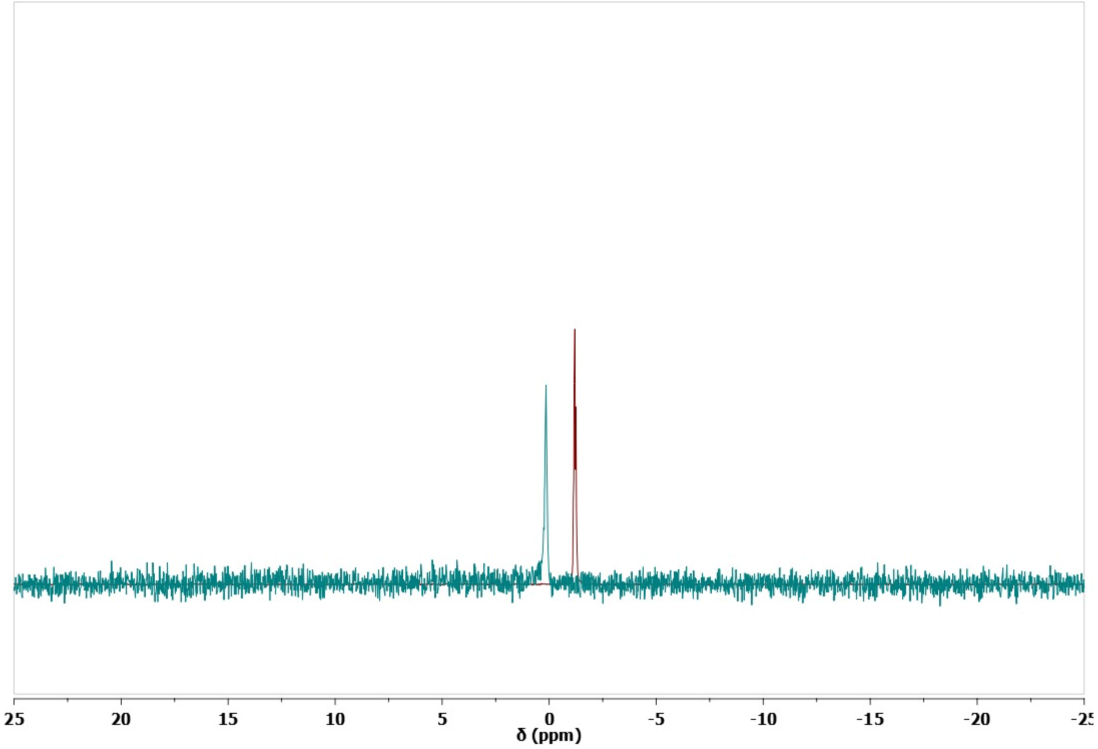

Figure S5. ^{31}P NMR spectra of PolyCOD$_{phosphonate}$ (red) and PolyCOD$_{phosphonic\ acid}$ (blue).

4.0 Vertical Burn Test

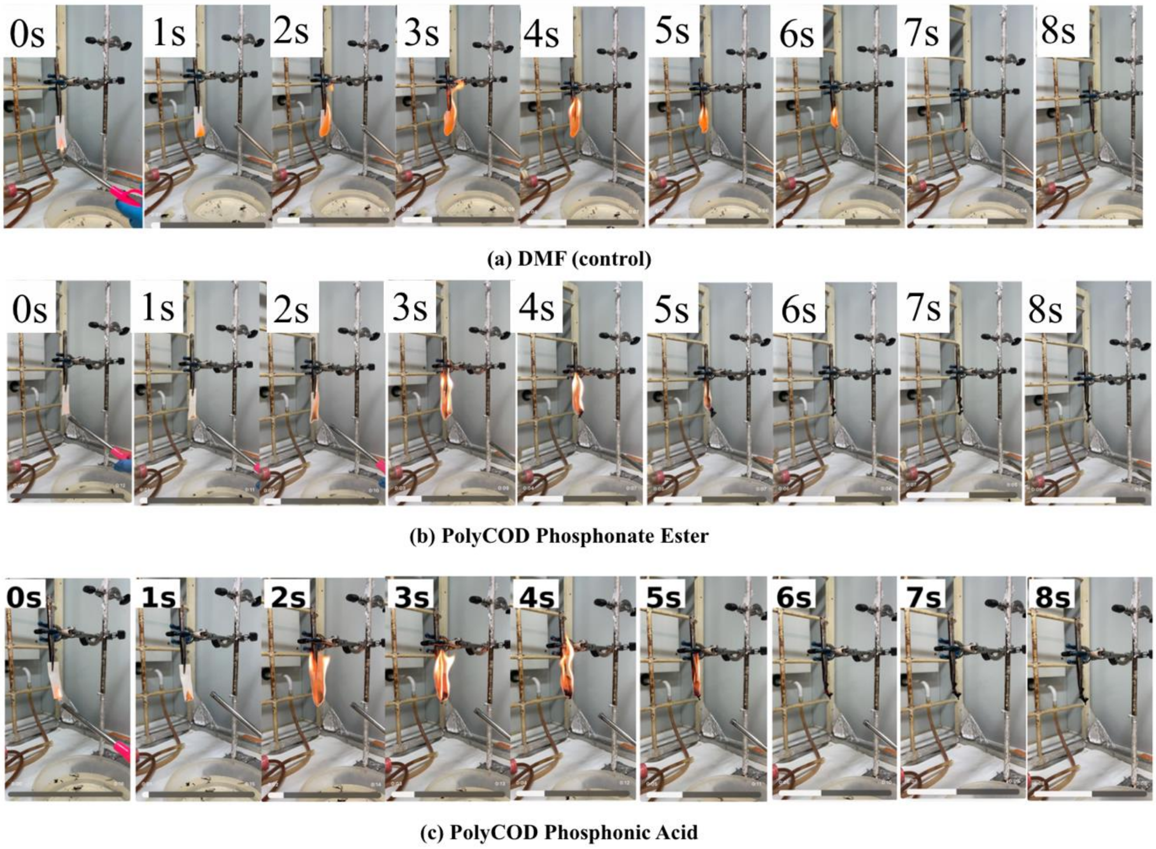

Figure S6. Photographs of the burn test conducted on uncoated filter paper (a), filter paper coated with PolyCOD phosphonate ester (b) and PolyCOD phosphonic acid polymers (c).

References

[1] J. A. Love, J. P. Morgan, T. M. Trnka, and R. H. Grubbs, “A Practical and Highly Active Ruthenium-Based Catalyst that Effects the Cross Metathesis of Acrylonitrile,” *Angewandte Chemie International Edition*, vol. 41, no. 21, pp. 4035–4037, Nov. 2002, doi: https://doi.org/10.1002/1521-3773(20021104)41:21<4035::AID-ANIE4035>3.0.CO;2-I.

[2] T. Eren and G. N. Tew, “Phosphonic acid-based amphiphilic diblock copolymers derived from ROMP,” *J Polym Sci A Polym Chem*, vol. 47, no. 15, pp. 3949–3956, Aug. 2009, doi: https://doi.org/10.1002/pola.23425.
